# A systematic literature review on the health-related quality of life and economic burden of Fabry disease

**DOI:** 10.1186/s13023-024-03131-y

**Published:** 2024-04-30

**Authors:** Ana Jovanovic, Eve Miller-Hodges, Felicia Castriota, Shweta Takyar, Heena Howitt, Olulade Ayodele

**Affiliations:** 1 The Mark Holland Metabolic Unit, Northern Care Alliance NHS Foundation Trust, Salford, UK; 2 Centre for Cardiovascular Science, Queen’s Medical Research Institute, University of Edinburgh, Edinburgh, UK; 3 Takeda Development Center Americas, Cambridge, MA USA; 4 Parexel, Mohali, India; 5 Takeda UK Ltd, London, UK

**Keywords:** Fabry disease, Agalsidase alfa, Agalsidase beta, Migalastat, Health-related quality of life, SF-36, EQ-5D, Resource utilization, Cost burden, Health state utility values, Lysosomal storage disease

## Abstract

**Background:**

Fabry disease (FD) is a rare lysosomal storage disease associated with glycolipid accumulation that impacts multiple physiological systems. We conducted a systematic literature review (SLR) to characterize the humanistic (quality of life [QoL]) and economic burden of FD.

**Methods:**

Searches were conducted in the Embase, MEDLINE^®^, and MEDLINE^®^ In-Process databases from inception to January 19, 2022. Conference abstracts of specified congresses were manually searched. Additional searches were performed in the Cochrane and ProQuest databases for the humanistic SLR and the National Health Service Economic Evaluations Database for the economic SLR. Studies of patients with FD of any sex, race, and age, and published in the English language were included. There was no restriction on intervention or comparator. For the humanistic SLR, studies that reported utility data, database/registry-based studies, questionnaires/surveys, and cohort studies were included. For the economic SLR, studies reporting economic evaluations or assessing the cost of illness and resource use were included.

**Results:**

Of the 1363 records identified in the humanistic search, 36 studies were included. The most commonly used QoL assessments were the 36-item Short-Form Health Survey (n = 16), EQ-5D questionnaire descriptive system or visual analog scale (n = 9), and the Brief Pain Inventory (n = 8). Reduced QoL was reported in patients with FD compared with healthy populations across multiple domains, including pain, physical functioning, and depressive symptoms. Multiple variables—including sex, age, disease severity, and treatment status—impacted QoL. Of the 711 records identified in the economic burden search, 18 studies were included. FD was associated with high cost and healthcare resource use. Contributors to the cost burden included enzyme replacement therapy, healthcare, and social care. In the seven studies that reported health utility values, lower utility scores were generally associated with more complications (including cardiac, renal, and cerebrovascular morbidities) and with classical disease in males.

**Conclusion:**

FD remains associated with a high cost and healthcare resource use burden, and reduced QoL compared with healthy populations. Integrating information from QoL and economic assessments may help to identify interventions that are likely to be of most value to patients with FD.

**Supplementary Information:**

The online version contains supplementary material available at 10.1186/s13023-024-03131-y.

## Introduction

Fabry disease (FD; OMIM 301500) is a rare, multisystemic, X-linked inherited disorder of glycosphingolipid metabolism, which occurs as a result of decreased activity of the lysosomal enzyme α-galactosidase A (α-Gal A) [1–3]. The reduction in α-Gal A activity is caused by pathogenic variants in the α-Gal A gene (*GLA*) [1–3]. Functional α-Gal A breaks down glycolipids (i.e. globotriaosylceramide [GL-3 or Gb3], its deacylated form lyso-GL-3/Gb3 and related glycolipids). When normal α-Gal A activity is deficient, glycolipids accumulate across multiple physiological systems, including the renal, cardiovascular, and nervous systems [1–3].

There are two main phenotypes of FD: the more severe ‘classical’ form, and the variant ‘non-classical’ or ‘late-onset’ form [4]. As expected with an X-linked disease, both types of FD are more prevalent in men than in women [4]. Patients with classical FD have very low to no α-Gal A activity [5]; among patients with non-classical FD, there is greater heterogeneity in the level of α-Gal A activity [6]. The variation in α-Gal A activity levels in non-classical FD results in attenuated and variable disease presentation [6]. Early symptoms of classical FD include neuropathic pain, angiokeratomas, anhidrosis, and gastrointestinal symptoms [4]. The rate of disease progression of FD can vary considerably between patients [7].

The current standard of care for patients with FD is intravenous enzyme replacement therapy (ERT) with agalsidase alfa (Replagal^®^, Takeda Pharmaceuticals International AG) or agalsidase beta (Fabrazyme^®^, Sanofi Genzyme) [8–11]. Treatment with long-term ERT is costly and burdensome for patients [8, 12]. Therefore, treatment is generally limited to those at high risk or with evidence of major organ involvement. ERT is recommended for all adult male patients with classical FD [8, 9]. For those patients with an amenable *GLA* variant, oral chaperone therapy with migalastat (Galafold™, Amicus Therapeutics Inc.) can also be used for treatment [13]. ERT has been previously shown to have a positive impact on the quality of life (QoL) of patients with FD, as well as to preserve organ function, reducing cardiovascular and renal complication rates in patients who started treatment before the onset of irreversible organ damage [14, 15]. Early intervention with ERT in patients with FD may prevent progression to cardiovascular and end-stage renal disease (ESRD). However, current challenges include identifying patients who would benefit from early intervention and defining the optimum time of treatment initiation [16].

The overall burden of FD can be described as humanistic, given the number of progressively declining health issues that can directly affect patients’ QoL, and economic, given the high costs associated with managing the disease (treatment, hospitalizations, doctor visits, surgeries, diagnosis, and tests) and improving patients’ QoL. The importance of measuring QoL and capturing the humanistic impact of the disease (emotional, psychological, social, and physical function) is well documented in patients with FD [4]. A variety of questionnaires have been used to assess QoL in patients with FD, including the 36-item Short-Form Health Survey (SF-36), the EuroQol five dimension (EQ-5D) questionnaire, the 100-item World Health Organization Quality of Life scale (WHO QoL-100), the Brief Pain Inventory (BPI), and a visual analog scale (VAS) [4]. In a 2015 systematic review, Arends and colleagues described the negative impact of FD on patients’ QoL as assessed with the SF-36, EQ-5D, EQ-VAS, and BPI [4]. There are also assessments that focus on the mental well-being of patients, such as the Centre of Epidemiologic Studies Depression Scale (CES-D) and the Hospital Anxiety and Depression Scale (HADS) [17, 18], and scales to monitor impact on sleep, such as the Epworth Sleepiness Scale (ESS) [19]. Moreover, there are QoL assessments designed for pediatric populations, including the Pediatric Quality of Life Inventory (PedsQL), the Children’s Depression Inventory (CDI), the Behavior Assessment Scale for Children (BASC; available in multiple editions), and the Fabry-specific Pediatric Health and Pain Questionnaire (FPHPQ) [4, 20–22].

Health technology assessments (HTAs), required for reimbursement of therapies, tend to include some form of economic evaluation alongside clinical data of therapeutic benefit (notably, efficacy and safety data) [23]. Few countries include QoL, let alone health-related QoL, as criteria for reimbursement [23]. Previous research has also shown that traditional outputs for economic models may not be sensitive to the severity of rare diseases, potentially owing to the smaller populations with severe disease and the proportionally smaller improvements in health outcomes compared with those observed in larger, healthier populations [24].

Evidence synthesis is important for rare diseases such as FD, particularly given its wide phenotypic heterogeneity and relatively small study populations. Accordingly, we conducted a systematic literature review (SLR) to provide a comprehensive characterization of the current disease burden of FD, with focus on the humanistic impact on patients’ QoL (assessed with a broad range of tools) and the economic burden of disease (including healthcare resource utilization and costs). By reviewing these two aspects together, we aim to capture the overall burden of FD, both on patients and on wider society.

## Methods

This SLR was undertaken in accordance with the Preferred Reporting Items for Systematic Reviews and Meta-Analysis (PRISMA) guidelines [25]. Two separate searches were conducted to look at humanistic burden (with a focus on QoL) and economic burden (with a focus on healthcare resource utilization, costs, and economic evaluations). Key biomedical electronic literature databases were searched from inception to January 19, 2022. Embase^®^, MEDLINE^®^, and MEDLINE^®^ In-Process were searched for both the humanistic and economic burden, using Embase^®^ and PubMed platforms; in addition, the National Health Service (NHS) Economic Evaluations Database (EED; via the Cochrane library interface and the EconLit database) was used for the economic search, and Cochrane and ProQuest were used for the humanistic burden search. The search strategy was formulated in accordance with the list of databases suggested by HTA agencies such as the National Institute for Health and Care Excellence (NICE) and the Scottish Medicines Consortium.

In addition to the database search, conference abstracts were hand searched to retrieve the latest studies. Relevant conferences for abstract screening included (for both searches unless otherwise stated): the Fabry Family Education Conference, the Lysosomal Diseases Conference, the European Conference on Recent Advances in Lysosomal Diseases, the International Society for Pharmacoeconomics and Outcomes Research (economic search only), the Society for the Study of Inborn Errors of Metabolism (SSIEM), and the We’re Organizing Research on Lysosomal Diseases (WORLD) Symposium. Additional economic sources were searched for outcomes and subgroups of interest that were not available in the publications, including the Food and Drug Administration (FDA), the European Medicines Agency (EMA), the National Institute for Health Research (NIHR)-HTA, and other HTA websites. Full details of both searches are provided in Additional file 1: Table 1.

Eligibility criteria are summarized in Additional file 1: Table 2. All results were limited to studies published in English. For both searches, all adults and children with a confirmed diagnosis of FD were included, and there was no restriction on intervention or comparator. For the humanistic burden evidence search, studies providing utility data, database/registry-based studies, questionnaires/surveys, and cohort studies (prospective/retrospective observational) were included. For the economic evidence search, studies reporting economic evaluations or assessing the cost of illness and resource use were included.

### Data collection and extraction

Two independent reviewers conducted the first screening of all titles and abstracts only, followed by a second screening based on full-text articles. Two independent reviewers also conducted the data extraction from each of the included studies. Any discrepancies between the decisions of the two reviewers at any stage were resolved by a third independent reviewer; overall, the third reviewer intervened to establish the inclusion of one publication [26] and the exclusion of three publications due to lack of relevant QoL data. If more than one publication was identified describing a single study, the data were compiled into a single entry in the data extraction table to avoid the multiple counting of patients and studies. Each publication was referenced in the table to recognize that more than one publication may have contributed to the entry.

### Outcome measures

Using a predefined extraction process, key data including study details, study characteristics, patient characteristics at baseline, QoL outcomes of interest, and resource utilization were recorded.

### Methodological appraisal

For the economic burden SLR, the quality of identified studies was evaluated using the Consolidated Health Economic Evaluation Reporting Standards (CHEERS) checklist, the Philips checklist, and the NICE single technology appraisal-adapted Drummond’s checklist. The CHEERS checklist, developed by the Professional Society for Health Economics and Outcomes Research (ISPOR) Health Economic Evaluation Publication Guidelines Reporting Practices Task Force, outlines a 24-item reporting guideline checklist to assess the overall reporting quality of economic evaluations [27]. The Philips checklist is recommended to inform critical appraisal of the quality of economic modeling study methods [28], and the adapted Drummond’s checklist critically appraises the methodology of cost burden and resource use studies [29].

## Results

### Humanistic burden SLR

#### Identified studies

**Fig. 1:**
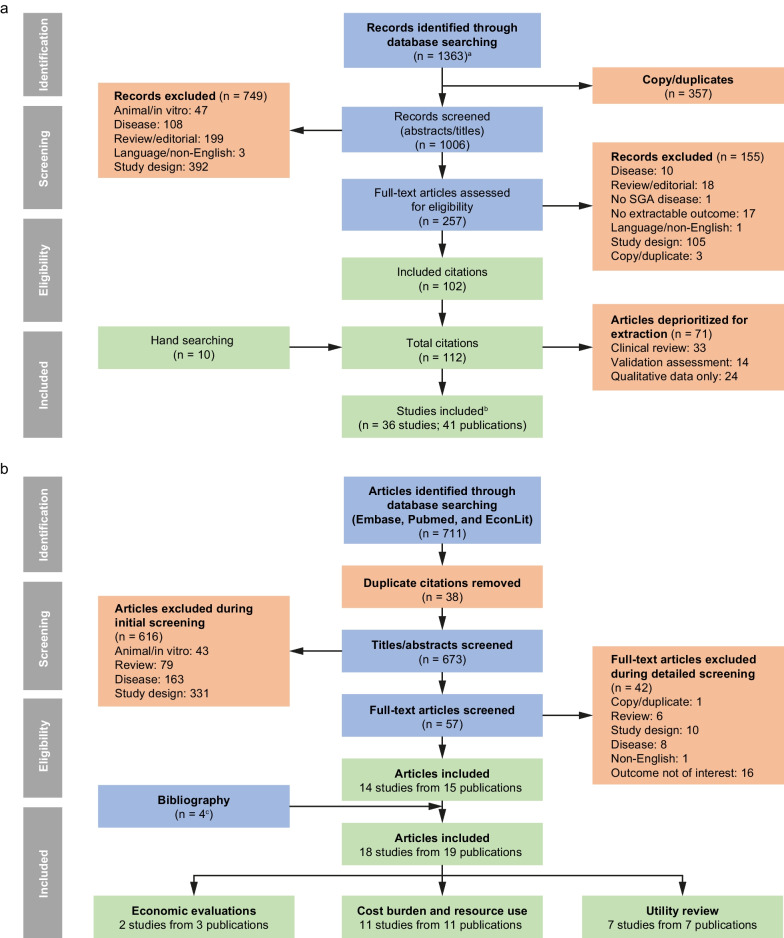
PRISMA flow diagram for the studies across the humanistic (**a**) and economic (**b**) searches. ^a^Databases included Embase^®^, MEDLINE^®^, Cochrane, PubMed and ProQuest. ProQuest was utilized for initial review conducted in January 2020; for the refresh of the current review (conducted in January 2022) only Embase^®^, Cochrane, and PubMed were utilized, in line with the protocol. ^b^In addition to the assessment of QoL, four studies from the economic burden SLR reported utility values with EQ-5D index scale. The EQ-5D results were included in the humanistic burden SLR and the utility values from these studies were included in the economic burden SLR. ^c^Four studies were retrieved from humanistic burden review as a part of bibliography. Some publications contained more than one economic study type; the numbers of publications by type are therefore not mutually exclusive. SGA, subgroup analysis

For the humanistic burden evidence, the initial electronic literature search identified 1363 records. Following the screening process, 36 studies (from 41 publications reporting QoL outcomes in patients with FD) were included in the analysis (Fig. 1A; Table 1A). Of the 36 included studies, 29 reported QoL outcomes in both male and female patients, of which eight studies reported data by sex. Four studies reported results only in female patients, and three studies reported results only in male patients. Three studies assessed a purely pediatric population.

**Table 1:** Summar﻿y of included studies from humanistic (A) and economic (B) searches

A											
Study reference	Publication type	Country	Study design	Study setting	Number of patients, N	QoL outcome measure	Patient population^a^	Patients analyzed, N	Age, years, mean (SD)	Age, years, median (range)	Proportion of female patients, %
Arends [45]	Journal article	Netherlands and UK	Cohort study	Multicenter	286	BPI, disease severity, ERT on EQ-5D utilities	Adult patients with FD	286	42.5 (15.5)	NR	60
							Adult male patients with classical FD	76	37.4 (12.5)	NR	0
							Adult male patients with non-classical FD	38	54.2 (15.4)	NR	0
							Adult female patients with classical FD	96	44.0 (15.5)	NR	100
							Adult female patients with non-classical FD	76	40.7 (15.2)	NR	100
Barba-Romero [46]	Journal article	Spain	Cross-sectional study	Multicenter	33	BPI, EQ-5D, NSS	Female patients with FD	33	45.6 (16.2)	44 (17–77)	100
Blackler [56]	Conference abstract	USA	Cohort (retrospective) study	NR	45	CES-D	Male and female patients with FD	45	NR	NR	57.7
Bouwman [30]	Journal article	Netherlands	Cross-sectional study	Single center	28	SF-36, CoL questionnaire	Male and female patients with FD	28	NR	NR	67.9
							Adult male patients with FD	9	NR	25 (18–35)	0
							Adult female patients with FD	19	NR	27 (18–33)	100
Bugescu [20]	Journal article	USA	Cohort study	Single center	24	PedsQL	Pediatric patients with FD	24	11.96 (3.2)	NR	58.3
Cazzorla [93]	Journal article	NR	Cohort study	NR	13	WHO QoL-100	Male and female patients with FD	13	49.6 (NR)	NR (33–67)	NR
Duning [58]	Conference abstract	NR	Survey (questionnaire)	NR	49	FSS	Male and female patients with FD	49	43 (NR)	NR	45.0
Gaisl [19]	Journal article	Switzerland	Cohort (prospective) study	Single center	52	ESS, SF-36	Male and female patients with FD	52	42.1 (14.2)	NR	67.3
Gibas [54]	Journal article	Canada	Survey	NR	552	NRS for pain	Male and female patients with FD	88	43.4 (13.2)	NR	58
							Male patients with FD	37	40.0 (12.1)	NR	0
							Female patients with FD	51	45.9 (13.5)	NR	100
Gold [31]	Journal article	USA	Survey	Single center	53	SF-36	Male patients with FD	NR	NR	NR	0
Hopkin [32]	Journal article	Global	Cohort study	Multicenter, international	352	BPI, SF-36	Male and female pediatric patients with FD	352	NR	NR	44.9
							Male pediatric patients with FD	194	11.4 (4.52)	12 (< 1–17)	0
							Female pediatric patients with FD	158	11.1 (4.43)	12 (< 1–17)	100
Ivleva [55]	Journal article	UK	Case–control study	Single center	154	Joint pain questionnaire	Male and female patients with FD and joint problems	77	51.8 (15.3)	54 (18–83)	63.6
							Controls without FD	77	49.5 (14.0)	52 (25–79)	63.6
							Male FD patients with joint problems	28	53.6 (16.4)	56.5 (18–80)	0
							Male controls without FD	28	50.0 (15.4)	53 (25–73)	0
							Female FD patients with joint problems	49	50.8 (14.7)	50 (21–83)	100
							Female controls without FD	49	49.3 (14.0)	50 (25–79)	100
Lachmann [26]	Conference abstract	Austria, Germany	Cohort study	Multicenter, international	114	SF-36	Male and female patients with FD	114	41.9 (16.02)	NR	56.1
Laney [94]	Journal article	USA	Cohort study	NR	30	ASEBA	Male and female patients with FD: clinical mean social-adaptive functioning deficit on ABCL or ASR (Yes)	8	37 (4.3)	NR	75
							Male and female patients with FD: clinical mean social-adaptive functioning deficit on ABCL or ASR (No)	22	41 (2.9)	NR	54.5
Lloyd [72]^b^	Journal article	UK	Survey	NR	506	Utility	Male and female patients with FD	506	46.93 (16.15)	NR	50.8
Loeb [57]	Journal article	Denmark	Cohort study	Single center	41	Neuropsychological tests, PDQ, HAM-D	Male and female patients with FD	41	47.2 (14.7)	NR (20–75)	71
Löhle [33]	Journal article	UK	Cross-sectional study	Single center	110 FD, 57 age-matched controls	BDI-II, RBDSQ, SF-36, BPI, EQ-5D VAS, ESS	Male and female patients with FD	110	49.0 (16.0)	NR (17.3–84.4)	54.5
							Age-matched controls	57	48.3 (17.4)	NR (21.6–88.2)	50.9
							Male patients with FD	50	50.5 (15.9)	NR (19.0–81.2)	0
							Age-matched male controls	28	49.1 (17.1)	NR (23.7–86.3)	0
							Female patients with FD	60	47.8 (16.1)	NR (17.3–84.4)	100
							Age-matched female controls	29	47.5 (17.9)	NR (21.6–88.2)	100
Low [34]	Journal article	Australia	Cohort study	Single center	21	MMSE and NUCOG, EQ-5D VAS, SF-36	Male patients with FD	19	40.4 (11.9)	NR (20–62)	0
							Female patients with FD	2	NR	NR (20–56)	100
Miners [35]	Journal article	UK	Database registry	NR	38	EQ-5D descriptive system, SF-36	Male patients with FD	38	37.2 (9.2)	NR	0
Morier [51]	Journal article	USA	Cohort study^c^	NR	23	Patient health and lifestyle questionnaire	Male patients with FD	8	32.3 (11.3)	NR (18–46)	0
							Female patients with FD	15	26.9 (15.4)	NR (7–55)	100
Nowak [48]	Journal article^d^	Germany, Switzerland	Cross-sectional study	Multicenter	124	EQ-5D, VAS	Male patients with FD	52	NR	49 (25–75)	0
							Female patients with FD	72	NR	48 (18–78)	100
Oder [36]	Journal article	Germany	Cohort (retrospective) study	Single center	6	SF-36	Male and female patients with FD	6	33 (15)	32 (18–51)	83
Pihlstrom [44]	Journal article	Norway	Cross-sectional study	NR	36	SF-36	Male and female patients with FD	36	49.1 (15.1)	NR	56
							Adult male patients with FD	16	50.2 (13.1)	NR	
							Adult female patients with FD	20	48.2 (16.8)	NR	
Polistena [47]	Journal article	Italy	Cross-sectional study	NR	106	EQ-5D	Male and female patients with FD	106	42 (NR)	NR (5–77)	59
							Male patients with FD	43	37 (NR)	NR (7–67)	0
							Female patients with FD	63	45 (NR)	NR (5–77)	100
Ries [50]	Journal article	USA	Cross-sectional study	Single center	25	CHQ	Pediatric male patients with FD	25	12.3 (3.5)	NR	0
Rosa Neto [37]	Journal article	Brazil	Survey	NR	37	SF-36, BPI	Male and female patients with classical FD	37	43.1 (15.4)	NR	57
Santamaria [49]	Conference abstract	NR	Cross-sectional study	NR	42	BPI, HADS, EQ-5D VAS	Patients with late-onset FD	42	44.43 (17.92)	NR	52.3
Sigurdardottir [43]	Journal article	Norway	Cohort study	Single center	43	SF-36	Male and female patients with FD	43	47 (15.3)	NR	53
							Male patients with FD	20	44.4 (8.8)	NR	0
							Female patients with FD	23	49.2 (19.2)	NR	100
Street [95]	Journal article	Global	Cohort study	Multicenter	202	RAND-36	Heterozygous female patients with FD	202	NR	NR (35–44)	100
Torvin Møller [53]	Journal article	NR	Case–control study	NR	19	VAS score for pain	Female patients with FD	19	NR	NR (18–65)	100
Wadley [42]	Journal article	USA	Case–control study	NR	270	SF-12, CES-D-4, PSS-4	Male and female patients with FD aged ≥ 45 years	54	55.7 (6.7)	NR	69
							Controls without FD	216	56.0 (6.6)	NR	69
Wagner [38]	Journal article	Germany	Cohort study	Single center	96	SF-36, VAS for pain	Male and female patients with FD and eGFR ≥ 60	77	38.8 (13.1)	NR	60.9
							Patients with FD and eGFR < 60	10	53.0 (11.3)	NR	40.0
							Patients with RRT	9	37.0 (8.6)	NR	0
Wang [52]	Journal article	USA	Cohort study	Single center	44	BPI	Heterozygous female patients with FD	44	46.1 (17.4)	48 (NR)	100
Wilcox [39]	Journal article	USA	Cohort (retrospective) study	NR	2236	SF-36, BPI	Male and female patients with FD	2236	NR	NR	48.2
							Male patients with FD	1159	37.3 (14.9)	38.0 (1.0–82)	0
							Female patients with FD	1077	40.5 (17.4)	42.0 (0.0–86)	100
Wyatt [40]	HTA submission	UK	Cohort study	NR	311	SF-36, PedsQL	Male and female adult patients with FD	289	NR	NR	58.5
							Adult male patients with FD	120	44.9 (14.5)	44.5 (16.4–78.6)	0
							Adult female patients with FD	169	43.9 (15.1)	43.7 (16.2–75.4)	100
							Male and female pediatric patients with FD	22	NR	NR	50.0
							Male pediatric patients with FD	11	8.9 (4.1)	9.1 (1.8–14.6)	0
							Female pediatric patients with FD	11	11.6 (3.3)	12.1 (4.6–15.9)	100
Żuraw [41]	Journal article	Poland	Cross-sectional study	Single center	33	EQ-5D descriptive system, SF-36, self-perception of health status (‘author’s questionnaire’)	Male and female patients with FD	33	31 (15)	NR	39.0

B								
Study reference	Publication type	Country	Study design	Patient population	Cost year	Number of patients, N	Time frame	Outcomes
***Economic evaluations***								
Inoue [60]	Conference abstract	Japan	Cost minimization (public health and societal perspective): migalastat vs ERT (agalsidase alfa/beta)	Overall FD cohort	2009	NA	Lifetime (cycle length 1 year)	Total medical cost, QALY, YFEOD, ICER
Rombach [59]	Journal article	Netherlands	Cost-effectiveness analysis (societal perspective): ERT vs standard medical care	Overall FD cohort	NR	NA	Lifetime (cycle length 1 year)	Total lifetime cost per patient
***Cost burden and resource use***								
Guest [63]	Journal article	Norway	Budget impact study	Adults with FD	2008/2009	64	1 year	Total annual cost per patient Hospital visits
Guest [64]	Journal article	Italy	Budget impact study	Adults with FD	2008/2009	240	1 year	Total annual cost per patient Hospital visits
Hilz [70]	Conference abstract	Germany	Retrospective cohort study	Male and female patients with FD	NA	46	8 years	Outpatient care, hospital care, sick leaves
Londono [67]	Conference abstract	Colombia	Cost analysis	FD	2019	1139	NR	Mean annual cost
Manrique-Rodríguez [62]	Journal article	Spain	Retrospective study	FD	NR	11	4 years	Cost saving of ERT
Meghji [69]	Journal article	USA	Retrospective study	Male and female adult patients with FD	NA	7	7 years	Hospital length of stay
Moore [68]	Conference abstract	NR	Cost-utility analysis	FD	NR	NR	1 year	Cost of ERT
Pinto [65]	Conference abstract	Colombia	Simulation model study	FD	NR	NR	15 years	Total cost of the cohort
Santamaria [61]	Conference abstract	Spain	Cross-sectional study	Patients with FD aged > 14 years old	NR	42	1 year	Hospital, non-hospital and pharmacological cost, healthcare resource use, productivity loss
Wallace [66]	Conference abstract	USA	Retrospective cohort study	Male and female patients with FD aged ≤ 65 years of age	NR	1705	6 years	Annual cost and medical cost ED visits, physician office visits, outpatient hospital visits, prescription medications
Wyatt [40]	HTA report	UK	Cost of illness analysis/HTA report	Adults and children with FD	2011	311	1 year	Hospital, non-hospital and social care cost Health and social care resource use; QALYs
***Utility review***								
Arends [45]	Journal article	Netherlands and UK	Cohort study	Adult male and female patients with FD	NA	286	NA	Utility scores (EQ-5D)
Lloyd [72]	Journal article	UK	Survey (discrete choice experiment)	Male and female patients with FD	NA	–	NA	Disutility scores
Miners [35]	Journal article	UK	Database registry	Male patients with FD	NA	38	NA	Utility scores (EQ-5D)
Nowak [48]	Journal article	Germany, Switzerland	Cross-sectional study	Male and female patients with FD	NA	124	NA	Utility scores (EQ-5D)
Polistena [47]	Journal article	Italy	Cross-sectional study	Male and female patients with FD	NA	106	NA	Utility scores (VAS)
Rombach [59]	Journal article	Netherlands	Cost-effectiveness analysis	Overall FD cohort	NA	97	NA	Utility scores (based on time trade-off, EQ-5D)
Wyatt [40]	HTA submission	UK	Cohort study	Adults and children with FD	NA	–	NA	Utility scores (EQ-5D)

^a^Please note: not all QoL outcomes are presented for all subgroups

^b^This study was identified as part of the humanistic search although is discussed in the context of the economic burden analysis only

^c^All 23 patients in this study belonged to a single family

^d^This preprint article has not undergone peer review

ASEBA, Achenbach System of Empirically Based Assessment; ABCL, adult behavior checklist; ASR, adult self-report; BDI-II, Beck Depression Inventory-II; BPI, Brief Pain Inventory; CES-D, Center for Epidemiologic Studies Depression Scale; CHQ, Child Health Questionnaire; CoL, course of life; ED, emergency department; eGFR, estimated glomerular filtration rate; EQ-5D, EuroQol five dimension; ERT, enzyme replacement therapy; ESS, Epworth Sleepiness Scale; FD, Fabry disease; FSS, Fatigue Severity Scale; HADS, Hospital Anxiety and Depression Scale; HAM-D, Hamilton Rating Scale for Depression; HTA, health technology assessment; ICER, incremental cost-effectiveness ratio; MMSE, Mini Mental State Examination; NA, not applicable; NR, not reported; NRS, Numerical Rating Scale; NSS, Neuropathic Sensory Symptom; NUCOG, Neuropsychiatric Unit Cognitive Screen; PDQ, Perceived Deficits Questionnaire; PedsQL, Pediatric Quality of Life Inventory; PSS-4, Perceived Stress Scale – 4 items; QALY, quality-adjusted life-year; QoL, quality of life; RBDSQ, Rapid Eye Movement Sleep Behavior Disorder Screening Questionnaire; RRT, renal replacement therapy; SD, standard deviation; SSF-12, 12-item Short-Form Health Survey; SF-36, 36-Item Short-Form Health Survey; VAS, visual analog scale; WHO, World Health Organization, YFEOD, years free of end-organ damage

Identified studies were conducted at both a global and country level and varied by study type. Nine studies were conducted in the USA, five studies were conducted in the UK, two studies each were conducted in Germany and Norway, and one study each was conducted in Australia, Brazil, Canada, Denmark, Italy, Netherlands, Poland, Spain, and Switzerland. Two studies reported data globally, while study country was not reported in four studies. Three studies were conducted in two countries (one in Germany and Austria, one in Germany and Switzerland, and one in the Netherlands and the UK).

Half of the studies in this analysis were cohort studies (n = 18), and the remaining studies were cross-sectional (n = 9), registry-/survey-based studies (n = 6), or case–control studies (n = 3). The sample size across the included studies ranged from 6 to 2236 patients. There were 32 studies that reported a mean/median age, which ranged from 8.9 to 56.5 years.

In total, 25 different instruments were used to assess QoL across the included studies. These instruments included general QoL scales and specific symptom scales; descriptions of the tools and a summary of their use across studies are provided in Additional file 1: Table 3. SF-36, BPI, and EQ-5D were used most often across the identified studies.

Normative populations refer to the general population or healthy controls, as applicable; these populations were included for comparison with patients with FD.

#### Impact of FD on overall QoL

##### QoL findings from studies using SF-36 and the 12-item Short-Form Health Survey (SF-12)

The SF-36 questionnaire is a generic instrument used to measure health-related QoL, with 36 questions covering aspects of physical and psychological functioning (each domain is scored from 0 [worst] to 100 [best]). The SF-12 is a shortened version of the SF-36 questionnaire, covering the same domains. SF-36 was used in 16 studies [19, 26, 30–44].

###### SF-36 scores in FD compared with normative populations

In 9 of the 16 studies, data were available comparing SF-36 scores in the reported FD population to a relevant normative population (Table 2). Overall, when compared with the general population or healthy controls, reduced QoL was reported among patients with FD across a range of domains, with some studies reporting a significant impact across every domain studied. Among those studies with statistical significance of differences calculated, physical functioning, bodily pain, and general health perception were the domains most frequently affected. No statistical comparisons were reported between patients with FD and age-matched controls.

**Table 2:** Summary of SF-36 domain scores in patients with FD compared with normative populations

Study	Comparative groups and *p* values	Number of patients, n	SF-36 total score, mean (SD)	Physical component summary score, mean (SD)	Mental component summary score, mean (SD)	SF-36 domain scores				
						Physical functioning, mean (SD)	Physical functioning, median (range)	Physical role limitations, mean (SD)	Physical role limitations, median (range)	Bodily pain, mean (SD)
Bouwman [30]	Men with FD	9	NR	NR	NR	NR	70 (50–95)	NR	100 (0–100)	NR
	Male controls	239	NR	NR	NR	NR	97.3 (NR)	NR	90.6 (NR)	NR
	*p* value (NS if *p* > 0.05)	–	–	–	–	–	0.007	–	NS	–
	Women with FD	16	NR	NR	NR	NR	90 (55–100)	NR	100 (0–100)	NR
	Female controls	269	NR	NR	NR	NR	91.9 (NR)	NR	83.1 (NR)	NR
	*p* value (NS if *p* > 0.05)	–	–	–	–	–	NS	–	NS	–
Gaisl [19]	FD	52	NR	NR	NR	NR	90 (60–100)	NR	100 (50–100)	NR
	Control	104	NR	NR	NR	NR	100 (95–100)	NR	100 (100–100)	NR
	*p* value (NS if *p* > 0.05)	–	–	–	–	–	< 0.01	–	0.01	–
Gold [31]	Male patients with FD	53	NR	NR	NR	51.2 (29.5)	NR	26.9 (38.7)	NR	49.1 (23.6)
	General US male population	NR	NR	NR	NR	87.2 (21.3)	NR	86.6 (30.9)	NR	76.9 (23.0)
Löhle [33]	FD	110	65.2 (24.2)	NR	NR	NR	NR	NR	NR	NR
	Age-matched controls	57	85.4 (12.2)	NR	NR	NR	NR	NR	NR	NR
	Male patients with FD	50	68.2 (23.9)	NR	NR	NR	NR	NR	NR	NR
	Age-matched male controls	28	86.3 (12.0)	NR	NR	NR	NR	NR	NR	NR
	Female patients with FD	60	62.8 (24.4)	NR	NR	NR	NR	NR	NR	NR
	Age-matched female controls	29	84.6 (12.5)	NR	NR	NR	NR	NR	NR	NR
Low [34]	FD	NR	NR	NR	NR	72.8 (25.0)	NR	54.2 (40.5)	NR	63.2 (22.1)
	Control	NR	NR	NR	NR	92.5 (13.4)	NR	91.4 (23.2)	NR	86.4 (17.9)
	*p* value	–	–	–	–	< 0.01	–	< 0.01	–	< 0.01
Miners^a^ [35]	Male patients with FD	38	NR	35.5 (14.7)	41.5 (13.8)	65.6 (31.3)	NR	53.9 (45.9)	NR	55.8 (31.1)
	General UK male population	3727–4107	NR	50.9 (9.8)	52.1 (9.9)	89.6 (17.6)	NR	88.1 (27.7)	NR	84.1 (20.6)
	*p* value (NS if *p* > 0.05)	0.0001	–	0.0001	0.0001	0.0001	–	0.0001	–	0.0001
Pihlstrom [44]	Men with FD	16	NR	NR	NR	NR	63.7	NR	53.5	NR
	General Norwegian male population	917	NR	NR	NR	NR	88.1	NR	78.9	NR
	*p* value (NS if *p* ≥ 0.05)	–	–	–	–	–	0.001	–	0.007	–
	Women with FD	20	NR	NR	NR	NR	73.7	NR	62.2	NR
	General Norwegian female population	1091	NR	NR	NR	NR	84.9	NR	72.6	NR
	*p* value (NS if *p* ≥ 0.05)	–	–	–	–	–	0.148	–	0.341	–
Sigurdardottir^b^ [43]	FD	43	NR	NR	NR	71.7	NR	53.3	NR	51.0
	General Norwegian population	5396	NR	NR	NR	86.4	NR	76.6	NR	73.6
	*p* value	–	–	–	–	< 0.001	–	< 0.001	–	< 0.001
Żuraw^c^ [41]	FD	31	NR	NR	NR	43.7	NR	40.8	NR	35.6
	Male patients with FD	18	NR	NR	NR	40.5	NR	39.1	NR	37.7
	Female patients with FD	13	NR	NR	NR	48.1	NR	43.2	NR	32.5

Study	SF-36 domain scores										
	Bodily pain, median (range)	General health perceptions, mean (SD)	General health perceptions, median (range)	Energy/vitality, mean (SD)	Energy/vitality, median (range)	Social functioning, mean (SD)	Social functioning, median (range)	Emotional role limitations, mean (SD)	Emotional role limitations, median (range)	Mental health, mean (SD)	Mental health, median (range)
Bouwman [30]	52 (31–84)	NR	72 (20–90)	NR	65 (25–85)	NR	87.5 (50–100)	NR	100 (0–100)	NR	88 (40–100)
	87.8 (NR)	NR	80.4 (NR)	NR	74.5	NR	89.5 (NR)	NR	90.4 (NR)	NR	80.8 (NR)
	0.008	–	0.05	–	NS	–	NS	–	NS	–	NS
	74 (22–100)	NR	59.5 (20–100)	NR	47.5 (20–100)	NR	68.8 (50–100)	NR	100 (0–100)	NR	80 (44–100)
	78.9 (NR)	NR	78.3 (NR)	NR	67.4 (NR)	NR	85.9 (NR)	NR	81.1 (NR)	NR	76.4 (NR)
	NS	–	0.003	–	0.09	–	NS	–	NS	–	NS
Gaisl [19]	84 (62–100)	NR	72 (57–82)	NR	65 (50–75)	NR	100 (63–100)	NR	100 (67–100)	NR	80 (72–88)
	100 (72–100)	NR	82 (72–92)	NR	82 (72–92)	NR	100 (88–100)	NR	100 (100–100)	NR	80 (72–88)
	0.06	–	< 0.01	–	0.59	–	0.05	–	0.45	–	0.52
Gold [31]	NR	24.1 (22.5)	NR	31.4 (24.0)	NR	53.8 (28.4)	NR	52.9 (41.7)	NR	61.2 (21.7)	NR
	NR	73.5 (20.0)	NR	63.6 (20.0)	NR	85.2 (21.3)	NR	83.3 (31.3)	NR	76.4 (17.2)	NR
Löhle [33]	NR	NR	NR	NR	NR	NR	NR	NR	NR	NR	NR
	NR	NR	NR	NR	NR	NR	NR	NR	NR	NR	NR
	NR	NR	NR	NR	NR	NR	NR	NR	NR	NR	NR
	NR	NR	NR	NR	NR	NR	NR	NR	NR	NR	NR
	NR	NR	NR	NR	NR	NR	NR	NR	NR	NR	NR
	NR	NR	NR	NR	NR	NR	NR	NR	NR	NR	NR
Low [34]	NR	46.2 (25.6)	NR	45.8 (26.8)	NR	76.4 (31.2)	NR	77.8 (34.3)	NR	78.0 (22.1)	NR
	NR	78.8 (15.7)	NR	64.0 (18.2)	NR	91.3 (15.8)	NR	85.6 (29.3)	NR	75.4 (16.3)	NR
	–	< 0.01	–	< 0.01	–	< 0.01	–	0.26	–	0.49	–
Miners^a^ [35]	NR	37.6 (24.0)	NR	41.3 (24.4)	NR	57 (31.1)	NR	56.1 (47.8)	NR	60.7 (21.5)	NR
	NR	73.6 (19.4)	NR	63.5 (18.6)	NR	89.9 (18.3)	NR	85.8 (29.2)	NR	75.9 (16.2)	NR
	–	0.0001	–	0.0001	–	0.0001	–	0.0001	–	0.0001	–
Pihlstrom [44]	49.7	NR	43.7	NR	36.3	NR	54.7	NR	73.4	NR	70.6
	72.1	NR	73.4	NR	61.9	NR	89.0	NR	89.5	NR	81.9
	0.008	–	< 0.001	–	< 0.001	–	< 0.001	–	0.001	–	0.016
	56.1	NR	50.8	NR	45.0	NR	65.6	NR	100	NR	76.5
	66.9	NR	72.6	NR	57.2	NR	85.7	NR	87.4	NR	79.9
	0.220	–	< 0.001	–	0.059	–	0.004	–	NR	–	0.438
Sigurdadottir^b^ [43]	NR	49.0	NR	42.6	NR	62.8	NR	73.8	NR	74.4	NR
	NR	75.3	NR	60.7	NR	86.3	NR	84.2	NR	80.2	NR
	–	< 0.001	–	< 0.001	–	< 0.001	–	< 0.05	–	< 0.05	–
Żuraw^c^ [41]	NR	42.9	NR	42.8	NR	29.0	NR	43.1	NR	38.4	NR
	NR	45.1	NR	42.7	NR	28.6	NR	44.1	NR	38.5	NR
	NR	39.8	NR	42.8	NR	29.6	NR	41.7	NR	38.3	NR

^a^Number of patients for the ‘general UK population’ comparator group ranged between 3727 and 4107 depending on the specific domain

^b^Baseline values from study presented here

^c^Norm-based scores presented, using data from the Swedish general population (n = 8930)

FD, Fabry disease; NR, not reported; NS, not significant; SD, standard deviation; SF-36; 36-item Short-Form Health Survey

###### Impact of patients’ sex on SF-36 scores

Seven studies analyzed SF-36 scores in both male and female patients, with two performing a direct statistical comparison between sexes [30, 32, 33, 37, 39, 41, 44]. In the study by Rosa Neto and colleagues, only scores in the general health perception domain differed significantly between male and female patients (female mean [standard deviation, SD], 56.4 [20.7]; male mean [SD], 39.1 [17.1]; *p* = 0.01) [37]. In the study by Pihlstrom and colleagues, however, only the emotional role domain differed significantly (female median [interquartile range], 100 [22.9]; male mean [SD], 73.4 [18.1]; *p* = 0.012) [44]. Overall, SF-36 scores were generally lower in male patients compared with female patients.

###### Impact of patients’ age on SF-36 scores

Two studies evaluated the impact of age on SF-36 scores [31, 39]. Wilcox and colleagues presented findings across six age groups (youngest 18–24 years; oldest ≥ 65 years), highlighting that, for both male and female patients, the most significant differences compared with the relevant normative population were among those aged 35–55 years (≥ 7 of 8 domains significantly different) [39]. Generally, while male patients had lower mean scores than female patients at a younger age, female patients experienced a greater decline in scores over time than male patients [39]. In the study by Gold and colleagues, patients were categorized into three age groups (< 20 years, 20–40 years, and > 40 years); SF-36 domain scores generally decreased with age, with the largest decreases observed between the 20–40 years and the older than 40 years age groups [31].

###### Impact of kidney function impairment on SF-36 scores

One study, by Wagner and colleagues, stratified SF-36 scores according to kidney function based on three groups: estimated glomerular filtration rate (eGFR) 60 mL/min or higher (preserved), eGFR below 60 mL/min (moderately impaired), and patients receiving renal replacement therapy (RRT) (severely impaired) [38]. Significant differences were observed between the groups across all SF-36 domains; physical domains were affected even in patients with moderately impaired kidney function, whereas an impact on mental/emotional and physical domains was observed mainly in those with severely impaired function.

###### Impact of length of treatment with ERT on SF-36 scores

Three studies evaluated how SF-36 scores are affected by the length of time a patient has received ERT [26, 34, 40]. In a longitudinal cohort study that recruited 311 patients, Wyatt and colleagues reported that, after adjusting for age, patients who had received ERT for more than 3 years had significantly lower scores than those who had been treated for up to 3 years [40]. In contrast, in a cross-sectional study, Low and colleagues found no significant changes in any domain scores over 21 months of follow-up (n = 40) [34]. The studies by Wyatt and colleagues and Low and colleagues, however, differed in both sample size and follow-up time, which may provide an explanation for the contrasting conclusions. Owing to the progressive nature of FD, different treatment lengths and follow-up time are likely to considerably affect results. The study by Lachmann and colleagues focused on home treatment with ERT and found that both physical and mental component summary scores increased following the switch from clinic-based infusions to home-based infusions [26]. No studies were identified that evaluated how SF-36 scores are affected by time receiving oral chaperone therapy. A fourth study by Sigurdardottir and colleagues found that SF-36 scores remained unchanged over a 7–13-year follow-up in a mixed population of male and female patients receiving ERT or chaperone or neither therapy. However, this study did not assess the effect of therapy on SF-36 score [43].

###### SF-12 findings

One study used SF-12—a shorter questionnaire than SF-36—to assess QoL in patients with FD aged 45 years or older [42]. Physical and mental component scores were significantly lower in patients with FD compared with controls.

##### Impact of FD on QoL as measured by EQ-5D

###### EQ-5D descriptive system findings

**Fig. 2:**
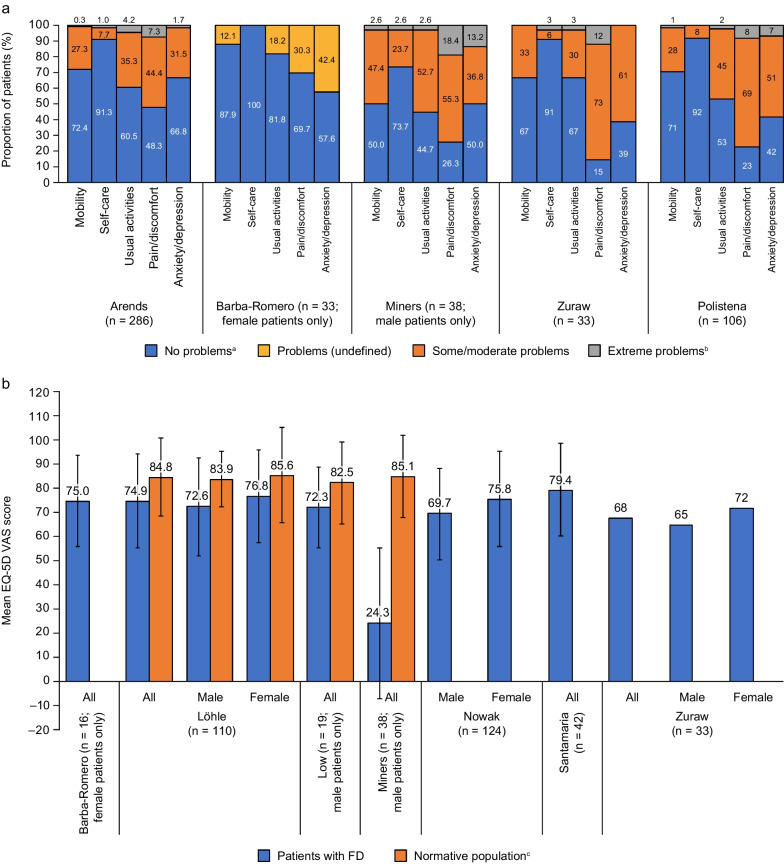
EQ-5D findings from studies using the descriptive system (**a**) and the VAS (**b**). ^a^The proportion of patients with ‘no problems’ was calculated using a subtractive approach based on the proportions of patients with ‘moderate problems’ or ‘extreme problems’ for Żuraw et al. [41]. ^b^‘Extreme problems’ was expressed as ‘confined to bed’ for all dimensions in in Miners et al. [35] and for the mobility dimension in Polistena et al. [47]. ^c^No normative population comparator was included for Barba-Romero et al. [46] or Żuraw et al. [41]. Data obtained from Arends et al. [45], Barba-Romero et al. [46], Löhle et al. [33], Low et al. [34], Miners et al. [35], Nowak et al. [48], Polistena et al. [47], Santamaria et al. [49], and Żuraw et al. [41]. Error bars represent standard deviations, where reported

The EQ-5D descriptive system asks respondents to report the extent of the problems they experience across five different dimensions. Five studies evaluated the impact of FD on QoL in different patient populations using the EQ-5D descriptive system [35, 41, 45–47], and domain-specific findings are presented in Fig. 2A. In nearly all studies, problems were reported by some patients in each domain evaluated. Pain/discomfort was the most frequently affected domain in all studies except the study by Barba-Romero and colleagues [46].

###### EQ-5D descriptive findings compared with normative population

The study by Miners and colleagues focused on EQ-5D descriptive findings in males with FD in the UK (n = 38), with comparison with a sample from the general UK male population (n = 1466) [35]. The proportion of patients experiencing problems was significantly higher than in the general population (*p* ≤ 0.001) across all dimensions.

###### Impact of patients’ sex and FD type on EQ-5D descriptive findings

Arends and colleagues evaluated EQ-5D results according to sex and diagnosis with classical or non-classical FD [45]. Among patients with classical disease [76 men and 76 women], a higher proportion of men than women experienced moderate or extreme problems in nearly all dimensions, with exceptions among patients with extreme problems with pain (9.4% of women compared with 5.3% of men) and anxiety/depression (similar proportions between sexes experiencing moderate problems, and no men or women experiencing extreme problems). Among those with non-classical disease (38 men and 76 women), the trend was less clear, with similar proportions of men and women experiencing moderate or extreme problems across dimensions, and women being more likely to experience extreme problems with self-care, usual activities, and pain and discomfort. Overall, the proportions of patients experiencing problems were similar between the classical and non-classical groups.

The prospective, cross-sectional study by Żuraw and colleagues, which included 20 men and 13 women, also identified that a higher proportion of men than women experienced problems across all dimensions, except anxiety/depression, with the biggest difference being observed in mobility (45% of men reporting moderate problems compared with 15% of women) [41].

###### Impact of ERT on EQ-5D descriptive findings

Żuraw and colleagues also looked at the impact of ERT on EQ-5D (n = 14 patients receiving ERT; n = 6 patients not on ERT) [41]. Higher proportions of patients with ‘any problems’ were reported across most dimensions in the ERT group; however, extreme problems were more common in the no ERT group (17%; [1 patient] with extreme problems in self-care and usual activities vs 0% in the ERT group; 50% [3 patients] with extreme pain/discomfort vs 7% [1 patient] in the ERT group).

###### Impact of health state on EQ-5D descriptive findings

Arends and colleagues evaluated the association of problems reported via the EQ-5D with health states [45]. Patients were asked to select from ‘no problems’, ‘some/moderate problems’, or ‘extreme problems’ for each EQ-5D domain. Among those patients with a relevant clinical event (neuropathic pain, organ involvement, ESRD, cerebrovascular accident, cardiac complications, or multiple complications), a higher proportion of patients experienced problems across most QoL domains compared with the asymptomatic group. For all dimensions except anxiety/depression, the patients with multiple complications (n = 18) were most likely to have experienced moderate or extreme problems.

###### EQ-5D VAS findings compared with normative population

The EQ-5D VAS rates QoL on a scale of 0 (worst imaginable health status) to 100 (best imaginable health status). Seven studies assessed QoL using this instrument [33–35, 41, 46, 48, 49]; one of these (Nowak and colleagues) is described in a preprint article [48]. An overview of mean VAS scores is presented in Fig. 2B. Mean VAS scores were generally similar for patients with FD across studies, ranging from 65–79, with the exception of the study by Miners and colleagues (a lower mean score of 24.3). Among the studies comparing scores to a relevant normative population, all reported a significantly lower mean score in the patient population compared with controls (*p* ≤ 0.02 in all three studies).

###### Impact of patients’ sex and ERT on EQ-5D VAS findings

Three studies evaluated EQ-5D VAS scores according to sex (Fig. 2B), each of which reported a numerically higher mean score in female patients than in male patients: 76.8 versus 72.6, 72 versus 65, and 75.8 versus 69.7 for females versus males across the three studies by Löhle, Żuraw, and Nowak, respectively, although none of these differences between sexes were statistically significant [33, 41, 48]. Żuraw and colleagues also analyzed the impact of ERT on EQ-5D VAS scores [41]. There was no difference between the ERT and no ERT groups, with a reported mean score of 65 in both groups. In contrast, Nowak and colleagues found that patients in the no ERT group (n = 24) had a significantly lower mean score compared with patients treated with ERT (n = 100) [48]. No studies were identified that analyzed the impact of oral chaperone therapy on EQ-5D VAS scores.

##### Impact of FD on QoL in children as measured by PedsQL

The PedsQL 4.0 instrument uses 23 items across four dimensions to evaluate QoL in children, giving a score from 0 to 100 (higher scores indicating better QoL). Two studies used the PedsQL 4.0 to assess QoL in children with FD [20, 40].

In the US case–control study performed by Bugescu and colleagues, children with FD self-reported significantly lower QoL than controls across all health dimensions, with the exception of emotional functioning; however, according to the parental report, QoL was lower across all domains (including emotional functioning) [20]. PedsQL scores were significantly lower in younger children than in adolescents (mean total score 68.20 vs 82.05; *p* = 0.007 for child self-report) and numerically higher in children receiving ERT compared with those not receiving ERT (difference not significant).

Wyatt and colleagues performed a longitudinal cohort study in the UK and evaluated the effect of treatment with ERT on PedsQL scores [40]. Scores were highest in all domains for patients not receiving ERT. Among those receiving ERT, scores were higher for patients treated for up to 3 years than for those treated for longer (statistical significance not calculated). However, PedsQL scores decreased significantly with age (*p* = 0.03) and, after adjusting for age, no relationship between time on ERT and any PedsQL subscale was observed.

##### Impact of FD on QoL in children as measured by Child Health Questionnaire (CHQ)

The CHQ is used to assess health-related QoL in children and adolescents, and includes both physical and psychosocial concepts. One study, by Ries and colleagues, used the CHQ to evaluate QoL in 25 male children with FD and 21 age-matched controls [50]. For patients younger than 10 years of age with FD (n = 9), mean QoL scores were numerically lower compared with controls (n = 212) across all aspects; however, only bodily pain and mental health scores were significantly different. For patients with FD and aged 10 years or older (n = 15), only the bodily pain score was significantly lower than the control value.

##### Additional QoL findings based on tools/instruments used in single studies

Several additional instruments were used to assess QoL in single studies identified in the literature review; the findings generally support a consistent picture of reduced QoL in patients with FD (Additional file 1: Table 4).

Two studies developed FD-specific instruments to assess QoL in patients [41, 51]. Żuraw and colleagues developed an ‘author’s questionnaire’ based on the literature, personal experiences, patient-related observations, and patient-collected information [41]. Self-perceived health status was evaluated, with some patients reporting ‘bad’ health status across all symptoms studied, most commonly for burning extremity pain (34%). After ERT, an improvement in symptoms was perceived for at least 50% of patients in each symptom category. An FD-specific questionnaire was also developed by Morier and colleagues (the ‘Patient Health and Lifestyle Questionnaire’) [51]. Most patients reported that FD impacted their QoL to some extent, with varying degrees of severity. An impact on QoL of any severity was reported by 87.5% of men compared with 60.0% of women. In comparison, the proportion of patients who reported that their life was greatly impacted was similar between men and women (12.5% vs 13.3%).

#### Impact of pain in FD

##### Pain in FD as measured using the BPI

The BPI assesses the severity of pain and the impact of pain on daily functions using scales of 1–10, with 10 being most severe or highest level of interference. A ‘pain severity index’ can be calculated as the arithmetic mean across the four severity items, and a ‘pain interference index’ as the arithmetic mean across the seven interference items.

###### BPI scores in FD overall and compared with normative populations

Eight studies reported BPI pain scores in FD; scores for those reporting data for the overall study population are summarized in Table 3 [32, 33, 37, 39, 45, 46, 49, 52]. Although all studies reported some degree of pain experienced by patients with FD, the severity and interference were variable within and between studies, and average pain scores tended to be towards the lower end of the scale (a BPI pain score < 5, which indicates mild pain). In the study by Löhle and colleagues, which compared findings to age-matched controls without FD, the average pain severity and interference with daily activities were significantly higher in patients than in controls (*p* = 0.002 and *p* = 0.003, respectively) [33].

**Table 3:** Summary of overall BPI scores and impact of sex, age, and disease subtype on BPI scores in patients with FD

Study	Comparative groups and *p* values (where applicable)	Number of patients, n	BPI pain severity score, mean (SD) overall	BPI pain severity score, median (range) overall	BPI interference score, mean (SD) overall	BPI interference score, median (range) overall	Impact of sex, age, and FD subtype: key results
Arends [45]	Adult male and female patients with FD	286	NR	2 (0–8)^a^	NR	0.6 (0–9.9)^a^	**Sex and FD subtype** In males, median scores for average and worst pain were higher in those with classical vs non-classical FD; in females, there were no differences in these scores between the FD subtype groups Interference score (median [range]) was: - higher in males with classical FD vs non-classical FD (0.5 [0–8.4] vs 0.1 [0–9.9]) - higher in females with non-classical FD vs classical FD (1.1 [0–9.7] vs 0.5 [0–9.3])
Barba-Romero [46]	Female patients with FD	28	6.9 (10)^b^	0 (0–29)^b^	1.2 (2.21)	0 (0–7.9)	NR
Hopkin [32]	Pediatric patients with FD	45	3.0 (2.59)^a,c^	3 (0–10)^a,c^	NR	NR	**Sex** BPI score for ‘worst pain in the past 24 h’ was significantly higher in males vs females (mean [SD], 4.4 [3.51]) vs 1.5 [2.45]; *p* < 0.02) No significant difference between the sexes in BPI score for average pain
Löhle [33]	Male and female patients with FD	110	1.9 (2.3)^d^	NR	1.7 (2.5)^d^	NR	**Sex** Females vs males had numerically higher scores for: - pain severity index (mean [SD], 2.2 [2.4] vs 1.5 [2.2]) - function interference index (mean [SD], 1.9 [2.5] vs 1.5 [2.6])
	Age-matched controls without FD	57	0.8 (1.5)^d^	NR	0.6 (1.3)^d^	NR	
	*p* value	NA	0.002	NA	0.003	NA	
Rosa Neto [37]	Patients with classical FD	37	2.78 (2.66), 2.80 (2.55) and 1.55 (2.38) for patients with severe, moderate and mild disease, respectively	NR	2.55 (2.44), 2.80 (3.18) and 1.36 (2.83) for patients with severe, moderate and mild disease, respectively	NR	NR
Wang [52]	Heterozygous female patients with FD	17–19 (depending on dimension)	2.9 (2.8)^a^	2 (NR)^a^	2.6 (3.3)^e^	1.5 (NR)^e^	NR
Wilcox [39]	Male and female patients with FD aged ≥ 12 years	2236	NR	NR	NR	NR	**Sex and age** Mean scores for average and worst pain were lower in females vs males in the 12–20- and 21–40-year age groups, but they were higher in females vs males among those aged > 40 years Mean ‘worst’ pain score in females increased from 1.5 in the 12–20-year age group to 3.4 in those aged > 40 years

^a^Pain on average

^b^Total pain or interference score

^c^Value reported is the first recorded score

^d^Recorded as severity or interference index

^e^Pain interference with general activity

BPI, Brief Pain Inventory; FD, Fabry disease; NA, not applicable; NR, not reported; SD, standard deviation

###### Impact of patient sex, age, and FD subtype on BPI findings

Hopkin and colleagues reported that the first recorded BPI score for ‘worst pain in the past 24 h’ was significantly higher in male versus female pediatric patients (aged 12–17 years; mean [SD], 4.4 [3.51] vs 1.5 [2.45]; *p* < 0.02), but they found no significant difference between sexes in the first recorded BPI score for average pain (Table 3) [32]. By contrast, Löhle and colleagues found that female patients, compared with male patients (all patients aged 17.3–84.4 years), had numerically higher pain severity index and function interference index [33]. The differences in the ages of the patient populations enrolled may explain the discrepancy between study findings. Wilcox and colleagues evaluated age and sex differences in BPI scores in patients aged 12 years and over [39]. In female patients, mean scores for average and worst pain were lower compared with in male patients up to the age of 40 years, but they were higher thereafter, suggesting a worsening of pain over time in female patients compared with relative stability in male patients. Arends and colleagues evaluated the effect of FD subtype and sex on BPI scores [45]. In male patients, median scores for average and worst pain were higher in those with classical versus non-classical disease, but this was not the case for female patients. Furthermore, the median interference score among males was higher for those with classical versus non-classical disease, while the opposite was true for females (higher for those with non-classical versus classical disease).

###### Impact of disease severity on BPI findings

Rosa Neto and colleagues compared BPI scores between patient groups classified according to FD severity [37]. Differences were limited between the ‘severe’ and ‘moderate’ groups, with the lowest scores observed in the ‘mild’ group. The mean (SD) BPI severity scores for patients with severe, moderate, and mild disease were 2.78 (2.66), 2.80 (2.55), and 1.55 (2.38), respectively, and the mean (SD) BPI interference scores were 2.55 (2.44), 2.80 (3.18), and 1.36 (2.83), respectively.

###### Impact of BPI score on QoL as assessed by the EQ-5D

Arends and colleagues included an evaluation of the relationship between BPI scores and EQ-5D utility scores [45]. Utilities significantly decreased with higher BPI scores, with an average 0.045 decrease in EQ-5D utility for every one-point increase in BPI average pain score (*p* < 0.001), indicating a relationship between increasing pain and worsening QoL for patients with FD.

##### Pain in FD as measured using a VAS for pain

Two studies used a VAS to assess pain, with a scoring system based on a range between 0 (no pain) and 10 (maximal pain) [38, 53]. Torvin Møller and colleagues assessed pain among female patients with FD in Denmark, reporting a median VAS score of 4.0 (range 1–7); 63% of patients noted that they experienced daily pain, and 42% reported pain crises within the past week [53]. A significant correlation was observed between age and VAS score in this population (*p* = 0.017). In the study by Wagner and colleagues, pain was assessed using the VAS according to chronic kidney disease (CKD) stages [38]. The median VAS score was similar across the three CKD groups: 2 (interquartile range, 1–3) in patients with eGFR 60 mL/min/1.73 m^2^ or higher, 2 (0–3) in patients with eGFR less than 60 mL/min/1.73 m^2^, and 2 (2–3) in patients receiving RRT.

##### Pain in FD as measured using the Numerical Rating Scale (NRS) for pain

Gibas and colleagues developed a questionnaire with 5-point scales for specific assessment of FD symptomatology, including pain [54]. Findings from the NRS questionnaire indicated significant variability between FD-related pain intensity and unpleasantness at its least, average, and worst (mean intensity ranging from 1.59 for ‘least’ pain to 4.43 for ‘worst’ in male patients and from 1.72 to 3.88 in female patients; mean unpleasantness ranging from 1.84 for ‘least’ pain to 4.38 for ‘worst’ pain in male patients and from 1.86 to 4.02 in female patients). Age was significantly correlated with FD-related pain at its worst for males (*p* < 0.05) but not for females. FD-related pain was rated as significantly more intense than other types of pain in patients overall, as well as in male and female subgroups.

##### Joint pain in FD as measured using the Joint Pain Questionnaire

One study used the Joint Pain Questionnaire to evaluate the impact of joint pain on daily life for patients with FD [55]. The proportion of patients with FD reporting current joint pain or swelling was higher than in age-matched controls (43.0% vs 25.0% for the male group and 39.0% vs 33.0% for the female group). Greater differences between patients and age-matched controls were observed when considering only those under 50 years of age (40.0% of male patients and 25.0% of female patients with current joint pain or swelling compared with 0% and 8.3% of age-matched male and female controls, respectively; *p* = 0.03 for the male comparison). There was also a higher proportion of patients overall than age-matched controls who had experienced joint swelling or joint pain lasting more than 4 continuous weeks (21.0% vs 14.0% for joint swelling, and 29.0% vs 14.0% for joint pain).

#### Mental health in FD

##### Depressive symptoms

Six studies evaluated depressive symptoms in patients with FD (Table 4) [20, 33, 42, 49, 56, 57]. Two studies used the CES-D, with scores ranging from 0 to 60 and higher scores indicating more depressive symptoms [42, 56]. In one of these studies by Blackler and colleagues, 42% of patients reported depressive symptoms, with 27% having severe symptoms; in the other study by Wadley and colleagues, there was a significantly higher proportion of patients with FD versus controls that had elevated depressive symptoms (28% vs 10%; *p* = 0.007). A study by Löhle and colleagues employed the Beck Depression Inventory-II (BDI-II) to assess depressive symptoms (higher scores indicating more severe depressive symptoms; maximum score 63) and reported a significantly higher mean BDI-II score in patients with FD versus age-matched controls, overall (9.8 vs 3.5; *p* < 0.0001) and in male (8.0 vs 3.2; *p* < 0.01) and female (11.3 vs 3.8; *p* < 0.01) subgroups [33]. In addition, the proportions of patients defined as having depression regardless of severity and of those defined as having severe depression were higher in patients with FD than in controls (26.8% vs 3.5% and 8.2% vs 0.0%, respectively; both *p* < 0.05).

**Table 4:** Summary of mental health assessments in FD

Study	Patient population (N)	Mental health symptoms assessed	Assessment tool	Key results
Blackler [56]	Male and female patients with FD (45)	Depression	CES-D (20-item scale to assess depressive symptom frequency over the previous week)	42% of patients reported depressive symptoms, 15% with mild to moderate symptoms and 27% with severe symptoms
Wadley [42]	Male and female patients with FD aged ≥ 45 years (54) Controls (216)	Depression	CES-D (4-item scale)	Elevated symptoms reported in a significantly higher proportion of patients with FD vs controls (28% vs 10%; *p* = 0.007)
		Stress	PSS-4	Scores were significantly higher, indicating greater perceived levels of stress, in patients with FD than in controls (mean [SD], 6.4 [2.3] vs 3.2 [2.7]; *p* < 0.0001)
Löhle [33]	Male and female patients with FD (110) Age-matched controls (57)	Depression	BDI-II	Significantly higher mean BDI-II scores in patients with FD vs age-matched controls: - overall, 9.8 vs 3.5; *p* < 0.0001 - male subgroup, 8.0 vs 3.2; *p* < 0.01 - female subgroup, 11.3 vs 3.8; *p* < 0.01 Significantly higher proportion of patients with depression (score ≥ 14; mild, moderate, or severe) (26.8% vs 3.5%; *p* < 0.05) and with severe depression (score ≥ 30; 8.2% vs 0.0%; *p* < 0.05) among patients with FD compared with controls
		Daytime sleepiness	ESS	Significantly higher mean ESS score, indicating greater daytime sleepiness, in patients with FD vs controls (7.2 vs 5.1; *p* = 0.009) A higher proportion of patients with FD reported significant sleepiness (score ≥ 10 points) compared with the control group (25.7% vs 19.3%) Findings in the male and female subgroups were similar to those for the overall population
		RBD	RBDSQ	Mean RBDSQ scores were comparable in patients with FD and the control group, indicating similar sleep behavior However, the proportion of patients reporting as RBD positive (score ≥ 5) was higher among those with FD compared with those in the control group (26.6% vs 14.0%)
Loeb [57]	Male and female patients with FD (41)	Depression	HAM-D	No significant differences in HAM-D scores between male and female patients, or between patients with and without cognitive impairment
Bugescu [20]	Pediatric patients with FD (24)	Depression	CDI-2	21% of patients with FD reported clinical levels of depressive symptoms (T score > 65) No significant differences in total depression or the CDI-2 subscales (emotional, interpersonal, functional problems, negative mood, negative self-esteem, and ineffectiveness) between patients with FD and previously established healthy samples
Santamaria [49]	Patients with late-onset FD (42)	Anxiety and depression	HADS	Anxiety prevalence, 45% Depression prevalence, 21%
Gaisl [19]	Male and female patients with FD (52)	Daytime sleepiness	ESS	Significantly higher mean ESS score, indicating greater daytime sleepiness, in patients with FD vs controls (7.6 vs 6.3; *p* = 0.01)
Dunning [58]	Male and female patients with mild to moderate FD (49)	Fatigue	FSS	Chronic fatigue prevalence, 45%

BDI-II, Beck Depression Inventory-II; CDI, Children’s Depression Inventory; CES-D, Center for Epidemiologic Studies Depression Scale; ESS, Epworth Sleepiness Scale; FD, Fabry disease; FSS, Fatigue Severity Scale; HADS, Hospital Anxiety and Depression Scale; HAM-D, Hamilton Rating Scale for Depression-17; PSS-4, Perceived Stress Scale – 4 items; RBD, Rapid eye movement sleep behavior disorder; RBDSQ, Rapid Eye Movement Sleep Behavior Disorder Screening Questionnaire; SD, standard deviation

Single studies assessed depressive symptoms in patients with FD based on the Hamilton Rating Scale for Depression-17 (HAM-D) and CDI [20, 57]. Loeb and colleagues found no significant differences in HAM-D scores between male and female patients or between those with and without cognitive impairment [57]. In the case–control study by Bugescu and colleagues, there were no significant differences in CDI-2 total score, scales, and subscales between patients with FD and previously established reference values for healthy individuals, although the small sample size (n = 24) may have impacted this result [20]. Despite this, 21% of patients with FD reported levels of depressive symptoms within the clinical range [20]. Another single study used the HADS and reported the prevalence of anxiety and of depression to be 45% and 21%, respectively, in patients with confirmed FD (aged > 14 years) [49].

##### Perceived stress levels, sleep, and fatigue

Wadley and colleagues reported a significantly higher score on the Perceived Stress Scale—4 items (PSS-4), indicating greater levels of perceived stress, in patients with FD compared with a control group (mean [SD], 6.2 [2.3] vs 3.2 [2.7]; *p* < 0.0001) (Table 4) [42]. Two studies, by Gaisl and colleagues and Löhle and colleagues, employed the ESS to assess average level of daytime sleepiness in patients with FD (a higher score corresponding to increased sleepiness; maximum score of 24); both showed greater daytime sleepiness in patients with FD compared with controls (7.6 vs 6.3 points [*p* = 0.01] and 7.2 vs 5.1 points [*p* = 0.009], respectively) [19, 33]. In addition, Löhle and colleagues reported that a higher proportion of patients with FD had significant sleepiness (score ≥ 10 points) compared with controls (25.7% vs 19.3%). Löhle and colleagues also performed an evaluation of features of rapid eye movement sleep behavior disorder (RBD) among patients with FD using the RBD Screening Questionnaire (RBDSQ) (Table 4) [33]; higher scores using this tool indicate more features associated with RBD (maximum score of 13). Comparable mean RBDSQ scores were reported for patients with FD compared with controls, indicating similar sleep behavior across the groups. There was, however, a higher proportion of patients with FD reporting as RBD positive (score ≥ 5) than of the control group (26.6% vs 14.0%).

In a study by Duning and colleagues, the prevalence of chronic fatigue in patients with mild to moderate FD, as assessed with the Fatigue Severity Scale (FSS), was 45% [58].

### Economic burden SLR

#### Identified studies

For the economic burden of evidence review, the initial electronic literature search identified 711 records. Following the screening process, 18 studies (from 19 publications) were included in the analysis; two studies (from three publications) reported economic evaluations, 11 (from 11 publications) reported cost burden and resource use, and seven (from seven publications) reported utility review (Fig. 1B; Table 1B). The two economic evaluation studies (one conducted in the Netherlands, and one in Japan) both included male and female patients.

Of the 11 studies reporting cost burden and resource use, two each were conducted in Colombia, Spain, and the USA, and one each in Germany, Italy, Norway, and the UK; one did not report a location. There were four retrospective studies, two budget-impact studies, a cross-sectional study, a simulation model study, a cost analysis, a cost-utility analysis, and a cost-of-illness analysis/HTA report.

Three of the studies reporting utility/disutility data were conducted only in the UK, one only in the Netherlands, one in the Netherlands and the UK, one in Italy, and one in Germany and Switzerland. There were two cohort studies, two cross-sectional studies, one cost-effectiveness analysis, one database registry study (conducted in males only), and one survey (discrete choice experiment).

Studies identified for each section were not mutually exclusive. One of the two economic evaluation studies also provided utility data; the remaining six utility studies were also identified in the humanistic burden SLR.

#### Economic evaluation of treatment for FD

The first of the two identified economic evaluation studies, performed by Rombach and colleagues, was a cost-effectiveness analysis in the Netherlands, comparing ERT with standard medical care from a societal perspective [59]. The second study, conducted by Inoue and colleagues, was a cost-minimization analysis performed in Japan, comparing migalastat with ERT (agalsidase alfa or beta) from both public healthcare and societal perspectives [60]. Both studies used a lifetime Markov state-transition model and a 1-year cycle length. Clinical data were obtained from various sources, including Phase 3 clinical trials, published literature, and the SEER database; cost data were sourced from published literature, medical records, official tariffs, and price lists; and resource use data were sourced from case reports and published literature [59, 60]. The Dutch study was based on 2009 costing with no discounting for the base-case (univariate analysis was restricted to the choice of discount rate to account for time preference: discounting of effects by 1.5% and costs by 4%) [59]. A discount rate of 2% was applied in the Japanese study; no cost year was reported [60].

The Dutch study indicated that, for patients with FD receiving ERT, the related incremental cost-effectiveness ratio (ICER) per quality-adjusted life-year (QALY), or per year free of end-organ damage (YFEOD) ranged from €3.2 million (discounted) to €6.5 million (without discounting) across the study [59]. ERT provided higher QALYs and YFEOD compared with no ERT (50.2 vs 48.6 and 56.5 vs 55.0, respectively). Total lifetime cost was lower with no ERT than with ERT (€270,964 vs €9,918,352). Incremental QALYs and YFEOD were larger for males than for females (1.7 vs 1.4 and 1.6 vs 1.3, respectively) favoring ERT as compared with no ERT. The incremental cost per additional YFEOD ranged from €5.9 million to €7.5 million, and the extra costs per additional QALY ranged from €5.5 million to €6.9 million, undiscounted [59]. In the assessment of reporting quality, this study met 22 of the 24 criteria on the CHEERS checklist [27], and 11 of the 17 criteria on the Philips checklist [28].

The Japanese study indicated that migalastat was associated with reduced costs when compared with ERT, from both the public health and the societal perspectives, driven primarily by savings in infusion-related costs [60]. In the base-case analysis (public healthcare payer), the total incremental lifetime cost per patient for ERT versus migalastat was JPY 90,193,830 (€700,846 [based on conversion rate on August 31, 2018; source: xe.com]) (JPY 780,140,002 [€6,062,034] for migalastat versus JPY 870,333,832 [€6 762 880] for ERT). Similarly, from the societal perspective, the total incremental lifetime cost per patient was JPY 94,440,730 (€733,846) (JPY 780,140,002 [€6,062,034] for migalastat vs JPY 874,580,732 [€6,795,880] for ERT). The sensitivity analyses confirmed the robustness of the results of the base-case analysis [60]. In the assessment of reporting quality, this study met 15 of the 24 criteria on the CHEERS checklist [27], and 8 of the 17 criteria on the Philips checklist; unmet criteria may have been attributable to the limitations of reporting in a conference abstract [28].

#### Cost burden and healthcare resource use associated with FD

##### Cost burden

Of the 11 studies identified in this section, nine reported cost burden data [40, 61–68]. An overview of the total costs associated with FD management and the contribution of ERT to those costs is presented by country in Table 5. The contribution of oral therapy to the total costs was not evaluated in these studies.

**Table 5:** Cost burden of FD management and HCRU by country

Country	Study	Total cost	ERT-related cost	Key HCRU data
UK	Wyatt [40]	**Estimated annual care costs of adult patients with FD, mean (SD)** All non-hospital NHS and social care providers: £1000 (£2702) Total health- (NHS) and social care cost: £3300 (£5958) **Estimated annual care costs of adult patients with FD, median (IQR)** All non-hospital NHS and social care providers: £81.50 (£16–340) Total health- (NHS) and social care cost: £1000 (£200–3200) **Estimated annual care costs of pediatric patients with FD, mean (SD)** All non-hospital NHS and social care providers: £710 (£1378) Total health- (NHS) and social care cost: £1300 (£1600) **Estimated annual care costs of pediatric patients with FD, median (IQR)** All non-hospital NHS and social care providers: £130 (£130–330) Total health- (NHS) and social care cost: £240 (£130–3200)	**Annual NHS cost per patient for adults** Agalsidase alfa^a^: £120,840 Agalsidase beta^b^: £106,394 **Annual NHS cost per patient for children** Agalsidase alfa^a^: £89,199 Agalsidase beta^b^: £79,478	**Use of hospital services among adults (n = 257) and children (n = 18)** 75% of adults; 39% of children **Use of non-hospital services among adults (n = 257) and children (n = 18)** 80% of adults; 94% of children During the analysis year, 94% of children recorded general practitioner visits, including home visits, compared with 72% of adults
Spain	Santamaria [61]	**Total expenditure, mean (SD)** Total: €50,991.45 (€82,012) MSSI < 20 subgroup: €35,184.53 (€73,168.06) MSSI 20–40 subgroup: €90,508.75 (€92,577.32)	**Pharmacological expenditure**^**c**^**, mean (SD)** Total: €47,461.28 (€81,685.85) MSSI < 20 subgroup: €33,107.81 (€72,895.93) MSSI 20–40 subgroup: €83,344.94 (€94,312.23)	**Hospital admissions** 7.14% of patients **Surgery related to FD** 14.3% of patients **Most common specialists visited** Nephrologists (100% of patients) Cardiologists (52.38% of patients) **Most frequently used diagnostic imaging techniques** Echocardiography (54.76%) Abdominal ultrasound (21.43%) **Mean (SD) productivity loss (daily and work activities)** Overall: loss of 3.28 (7.19) working days/year MSSI < 20 subgroup: loss of 1.69 (4.19) days/year MSSI 20–40 subgroup: loss of 8.7 (12.39) days/year
Spain	Manrique-Rodríguez [62]	NR	**Pharmacological expenditure per 70 kg adult patient** Average annual cost of agalsidase beta at the recommended dose of 1 mg/kg/2 weeks: approximately €155,000 **Cost saving over 4 years per patient by reducing agalsidase beta dose over time** €213,584 (up to 35% of the cost per patient)	
USA	Wallace [66]	**Annual cost, mean** FD with ESRD: US$98,461 FD with early-stage kidney disease: US$34,521 FD with CKD: US$52,281 FD without CKD: US$14,950	NR	**HCRU in patients with FD with (n = 341) vs without (n = 1549) CKD** **Mean annual emergency department visits** 0.77 vs 0.54; *p* < 0.01 **Mean annual physician office visits** 14.92 vs 9.75; *p* < 0.0001 **Mean annual outpatient hospital visits** 15.16 vs 3.41; *p* < 0.0001 **Mean annual prescription medications** 58.47 vs 22.81; *p* < 0.0001
USA	Meghji [69]	NR	NR	**Median (range) length of stay in hospital for patients with FD following septal myectomy** 5 (4–7) days
Norway	Guest [63]	**Expected cost per patient, first year following diagnosis (2008/2009 prices)** Patients not receiving ERT: NOK 158,691.00 Agalsidase alfa-treated patients (0.2 mg/kg): NOK 927,707.35 Agalsidase beta-treated patients (1 mg/kg): NOK 975,008.40 **Expected cost per patient, after first year following diagnosis (2008/2009 prices)** Patients not receiving ERT: NOK 80,910.00 Agalsidase alfa-treated patients (0.2 mg/kg): NOK 1,556,559.62 Agalsidase beta-treated patients (1 mg/kg): NOK 1,639,979.50	**Expected cost per patient, first year following diagnosis (2008/2009 prices)** Agalsidase alfa-treated patients (0.2 mg/kg): NOK 664,368.00 (72% of total) Agalsidase beta-treated patients (1 mg/kg): NOK 695,088.00 (71% of total) **Expected cost per patient, after first year following diagnosis (2008/2009 prices)** Agalsidase alfa-treated patients (0.2 mg/kg): NOK 1,439,464.00 (92% of total) Agalsidase beta-treated patients (1 mg/kg): NOK 1,506,024.00 (92% of total)	**Average number of attendances to the family practitioner’s office for infusions among patients with FD receiving ERT (n = 34) in an average year** 17.2
Italy	Guest [64]	**Expected cost per patient, first year following diagnosis (2008/2009 prices)** Patients not receiving ERT: €2836.00 Agalsidase alfa-treated patients (0.2 mg/kg): €115,384.00 Agalsidase beta-treated patients (1 mg/kg): €116,432.00 **Expected cost per patient, after first year following diagnosis (2008/2009 prices)** Patients not receiving ERT: €639.00 Agalsidase alfa-treated patients (0.2 mg/kg): €164,121.00 Agalsidase beta-treated patients (1 mg/kg): €165,635.00	**Expected cost per patient, first year following diagnosis (2008/2009 prices)** Agalsidase alfa-treated patients (0.2 mg/kg): €110,796.00 (96% of total) Agalsidase beta-treated patients (1 mg/kg): €110,796.00 (95% of total) **Expected cost per patient, after first year following diagnosis (2008/2009 prices)** Agalsidase alfa-treated patients (0.2 mg/kg): €160,040.00 (98% of total) Agalsidase beta-treated patients (1 mg/kg): €160,039.00 (97% of total)	**Average number of attendances to a hospital day ward for infusions among patients with FD receiving ERT (n = 175) in an average year** 25.7
Colombia	Pinto [65]	15-year total cost for FD: $84–92 million	Costs of ERT: > 95% of total cost Annual cost of ERT per patient (2008): approximately US$175,000	
Colombia	Londono [67]	NR	**Annual treatment cost in FD**^**d**^** (2019)** Patients receiving agalsidase alfa: US$141.137 million Patients receiving agalsidase beta: US$126.71 million	
NR	Moore [68]	NR	Annual cost of ERT estimated to be US$175,000–350,000 (upper and lower estimates based on current ERT costs, using skeptical and enthusiastic prior probabilities)	
Germany	Hilz [70]	NR	NR	**Outpatient specialist consultations post-index** Nephrologists (46%) Internal medicine physicians (44%) Ophthalmologists (28%) **Hospital stays post-index** At least one hospital stay post-index, 71% of patients Mean (SD) hospital stays post-index, 1.7 (1.7) **Hospital stays pre-index** At least one hospital stay pre-index, 20% of patients Mean (SD) hospital stays pre-index, 0.6 (1.4) **Mean duration of sick leave post- and pre-index,** 17 and 21 days, respectively

^a^Replagal^®^ 3.5 mg

^b^Fabrazyme^®^ 35 mg

^c^31% of patients received treatment with ERT or migalastat

^d^Treatment costs included treatment acquisition and stroke events associated with FD

CKD, chronic kidney disease; ERT, enzyme replacement therapy; ESRD, end-stage renal disease; FD, Fabry disease; HCRU, healthcare resource utilization; IQR, Interquartile range; MSSI, Mainz Severity Score Index; NHS, National Health Service; NR, not reported; SD, standard deviation

Overall, all studies that included ERT in a breakdown of overall FD-related expenditure identified ERT as a major contributor to the cost burden associated with FD across different countries [40, 61, 63–65], with a contribution of over 95% in some cases. In the Spanish study by Santamaria and colleagues, a majority of costs attributable to ERT was also observed in subgroups defined by lower (< 20) or higher [20–40] Mainz Severity Score Index (MSSI), although the costs were considerably higher in the latter group [61].

Guest and colleagues performed two similar studies—one in Norway and one in Italy—both of which found that the highest annual per-patient costs were expected in the first year after diagnosis for patients not on ERT; for patients receiving ERT, the estimated costs were higher in subsequent years [63, 64]. For patients not on ERT, diagnostic tests were the highest cost driver both in the first year following diagnosis and in subsequent years.

Other types of FD-related costs were reported and included those relating to healthcare and social care use (including visits to healthcare facilities and professionals), dialysis, and tests/procedures (including diagnostic tests). The relationship between time on ERT and healthcare costs was investigated in a study by Wyatt and colleagues [40]. No statistically significant association between time on ERT and total NHS social care cost, hospital care costs, or non-hospital care costs for patients with FD were observed.

Wallace and colleagues did not specifically consider the contribution of ERT to healthcare costs, but looked at overall FD-related expenditure according to presence or absence of CKD [66]. The study reported a 3.5-times higher mean annual cost for patients with CKD compared with patients without CKD (*p* < 0.01). Mean annual costs for patients with ESRD were 2.5-times higher than those for patients with earlier stages of kidney disease (*p* < 0.0001).

##### Healthcare resource use

Seven of the identified studies reported data relating to resource use by patients with FD (Table 5) [40, 61, 63, 64, 66, 69, 70].

In their 2012 study assessing healthcare resource use for patients with FD in the UK, Wyatt and colleagues found that the majority of adult patients used both hospital and non-hospital services; however, among pediatric patients, almost all used non-hospital services, but only 39% used hospital services. A higher proportion of pediatric than adult patients recorded general practitioner visits (including home visits) during the analysis year [40].

In a study by Santamaria and colleagues evaluating annual use of healthcare resources (hospitalization and surgeries, visits to health professionals, diagnostic tests, and treatments) in Spain, 7.14% of patients required admission and 14.3% required surgery related to FD [61]. The most common specialists visited were nephrologists and cardiologists; the most frequently used diagnostic imaging techniques were echocardiography and abdominal ultrasound. This study also calculated a mean (SD) productivity loss (daily and work activities) of 3.28 (7.19) working days/year in patients with FD overall, with the loss increasing with MSSI [61].

The two studies by Guest and colleagues estimated the resource implications of managing adults with FD in Norway and Italy, from the perspectives of the Norwegian publicly funded healthcare system and the Italian Servizio Sanitario Nazionale (SSN) [63, 64]. In an average year in Norway, patients receiving ERT were expected to make an average of 17.2 attendances to their family practitioner’s office for their infusions; in an average year in Italy, patients receiving ERT were expected to make 25.7 hospital attendances to a hospital day ward for infusions [63, 64].

In Germany, Hilz and colleagues quantified the burden of FD on patient productivity and healthcare utilization based on analysis of insurance claims [70]. Most patients received their first diagnosis in outpatient care. Specialists consulted in outpatient care post-index included nephrologists, internal medicine physicians, and ophthalmologists. Almost three-quarters of patients (71%) had at least one hospital stay post-index, while only a fifth of patients had at least one hospital stay pre-index. The number of hospital stays (mean [SD]) was also greater post-index (1.7 [1.7]) compared with pre-index (0.6 [1.4]). The mean duration of sick leave was 17 days post-index and 21 days pre-index [70].

A 2021 study by Wallace and colleagues demonstrated significantly higher healthcare resource utilization (including emergency department visits, physician office visits, outpatient hospital visits, and prescription medications) in patients with FD with versus without CKD [66].

###### Methodological appraisal of studies reporting cost burden and resource use data

Critical appraisal of cost burden and resource use studies was carried out using the adapted Drummond’s checklist as recommended in the NICE single technology appraisal manufacturer’s template [29, 71]. All 11 studies reported and discussed study results appropriately.

#### Health utility values in FD

Health state utility values measure preferences that patients attach to specific health-related outcomes, with a scale from 0.0 (death) to 1.0 (perfect health); they are often considered in health economics evaluations. Taken together, data from the seven studies providing utility values for FD (summarized in Table 6) demonstrated no clear effect of sex or age on utility values, but a decrease in utility with increasing number of complications, including cardiac, renal, and cerebrovascular morbidities [35, 40, 45, 47, 48, 59, 72]. Two studies by Arends and colleagues and Nowak and colleagues reported significantly lower utility values in patients with classical disease compared with those with non-classical disease (*p* = 0.037 [for males at age 50 years] and *p* < 0.01, respectively) [45, 48]. Furthermore, Arends and colleagues found no change in utility in patients who initiated ERT over a mean follow-up of 6.1 years [45].

**Table 6:** Utility (A) and disutility (B) values reported in patients with FD

A				
Study	Method/tool	FD group/health state	n	Mean utility value
Arends [45]	EQ-5D	Overall	286	0.77
		Men, classical	76	0.75
		Men, non-classical	38	0.81
		Women, classical	96	0.79
		Women, non-classical	76	0.76
		Before ERT	–	0.796^a^
		No organ involvement	31	0.851
		Organ involvement	221	0.78
		Neuropathic pain	21	0.725
		End-stage renal disease	7	0.83
		Cardiac complication(s)	16	0.705
		Multiple complications	45	0.732
		Cerebrovascular accident	18	0.530
Miners [35]	EQ-5D	Males	38	0.560
Nowak [48]	EQ-5D	Males	52	0.74
		Females	72	0.76
		Age > 40 years	–	0.72
		Age ≤ 40 years	–	0.72
		Classic	–	0.68
		Later-onset	–	0.82
		Kidney disease	46	0.69
		No kidney disease	78	0.79
		Heart disease	53	0.69
		No heart disease	71	0.81
Polistena [47]	VAS	Overall	106	0.65
		Men	63	0.63
		Women	43	0.66
Rombach [59]	Time trade-off	No symptoms	19	0.87
		Acroparesthesia/symptomatic	55	0.76
		Single complication	18	0.74
		Multiple complications	5	0.58
		Total	97	0.77
Wyatt [40]	EQ-5D	Age > 13 years	–	− 0.24 to 1.0^b^

B				
Study	Method/tool	FD group/health state	n	Estimated disutility value
Lloyd [72]	Disutility, by discrete choice experiment	Nurse-administered infusion (compared to oral tablet)	–	− 0.052
		Self-administered infusion (compared to oral tablet)	–	− 0.0543
		Reaction to your treatment 6 times a year (compared to no reaction)	–	− 0.0202
		Reaction to your treatment 12 times a year (compared to no reaction)	–	− 0.0361
		Headaches 6 times a year treatable with painkillers (compared to no headache)	–	− 0.0285
		Headaches 12 times a year treatable with painkillers (compared to no headache)	–	− 0.0473
		15% or under (1 in 7 people) will develop antibodies in a few years (compared to no antibodies)	–	− 0.0095
		25% or under (1 in 4 people) will develop antibodies in a few years (compared to no antibodies)	–	− 0.0278

^a^Median

^b^Range

EQ-5D, EuroQol five dimension; ERT, enzyme replacement therapy; FD, Fabry disease; VAS, visual analog scale

Lloyd and colleagues designed a discrete choice experiment to assess social preference weights for different features of FD treatments in the UK [72]. Participants (n = 506) were significantly more likely to choose a treatment associated with an increase in their life expectancy by 1 year (odds ratio, 1.574; 95% confidence interval CI 1.504–1.647) and significantly less likely to choose a self-administered intravenous treatment compared with an every-other-day tablet (odds ratio, 0.426; 95% CI 0.384–0.474). The estimated disutilities indicated that patients have a preference for an oral tablet over intravenous treatment, in terms of route of administration and avoidance of treatment infusion reactions, and a preference for treatments that are less likely to cause headaches—a potential side effect of some treatments for FD (Table 6).

## Discussion and conclusions

In recent years, there has been a shift in the focus of management of FD from treatment to prevention, with the aim of preserving organ function, preserving life expectancy, and optimizing QoL, and with a drive towards earlier diagnosis and management, as supported by consensus recommendations [7, 73–75]. Given the variable disease subtypes and relatively small clinical populations for rare diseases such as FD (global prevalence estimated at 1 in 40,000 to 1 in 170,000 [76]), clinical trial research can be challenging and traditional economic models may not be appropriate for evaluating the impact of treatments in these patients [7, 24, 73–75]. The aims of the present SLR were, therefore, to provide an update and broad overview of the current humanistic burden of FD (specifically the impact of FD on different measures of patients’ QoL) and of the current economic burden of FD (including healthcare resource utilization and costs). We find that FD still carries a substantial burden, in terms of QoL, healthcare resource use, and costs, indicating a significant unmet need in the management of FD. Moreover, QoL and health utility are impacted by factors such as sex, age, disease severity and complications, and treatment status.

Overall, a clear impact of FD on patients’ QoL was observed across the included studies; lower QoL scores were reported for patients with FD than healthy controls or the general population across multiple QoL domains [19, 30, 31, 33–35, 43, 44]. QoL was also influenced by variables such as sex, age, disease severity and manifestations, and treatment status. Generally, lower QoL scores were reported for men with FD than for women with FD [33, 37, 41, 44, 48], and QoL tended to decrease with increasing age [31, 39]. As an X-linked disease, FD is generally viewed as a disorder mainly affecting men; however, reduced QoL was reported for female patients with FD compared with healthy controls or the general population [33, 44], thus highlighting the importance of recognizing the substantial disease burden of FD in both sexes.

Alongside an increase in patient participation in treatment decisions and assessment of their own care, health-related QoL has become an increasingly important measure of treatment efficacy [77, 78]. As such, understanding the impact of disease on patients’ QoL is vital to understanding therapy effectiveness. Here, we found that there is a considerable range of instruments that are used to assess QoL in patients with FD: 25 different types of QoL assessment were identified, and the majority are not specific to FD. This finding highlights the need for standardization in the assessment of QoL in the form of an FD-specific QoL questionnaire. In agreement with Arends and colleagues [4], the findings of the present study indicate that an FD-specific assessment would be valuable for capturing the burden of disease and should include measures that form part of the SF-36 questionnaire, such as physical functioning and health perception, alongside pain, mental health, and sleep. Furthermore, an FD-specific assessment should be applicable or adaptable to both male and female adults with FD, in order to capture the broad and heterogeneous population that may be affected by this disease. Although a tool meeting all of these desired criteria has not yet been established, several recent studies (not captured in the current SLR search) have investigated new FD-specific tools, including: the Fabry Disease Patient-Reported Outcome—Gastrointestinal (FABPRO-GI) for assessing gastrointestinal signs and symptoms; the Fabry Disease—Patient-Reported Outcome (FD-PRO), which covers neuropathic symptoms, headache, abdominal pain, heat intolerance, swelling, tinnitus, fatigue, hearing/vision impairment, hypohidrosis, and difficulty engaging in regular physical activities in the past 24 h; a modified BPI—Short-Form item 3 (BPI-SF3) scale for assessing neuropathic pain specifically in patients with FD; and the Adult Fabry QoL Scale (AFQOL) comprising five domains—neuropathic pain and abdominal symptoms, impact on work and school, relationship challenges, ophthalmologic and otolaryngologic symptoms, and cardiovascular and renal symptoms [79–82].

Health utility values were typically lower in patients with classical FD than in those with non-classical FD [45, 48]. Moreover, health utility values were influenced by severity of disease and number of complications; utility decreased with increased disease complications [45, 48, 59]. Although age, sex, and ERT status influenced QoL scores, no clear association was reported between these factors and health utility values.

In terms of the economic burden, FD was associated with a high cost and healthcare resource use burden [40, 61, 64, 66, 67]. All studies that included patients who were receiving ERT reported that it made a substantial contribution to the cost of FD management [40, 61, 63–65]. In a Japanese study, migalastat was associated with lower costs than ERT, primarily driven by savings in infusion-related costs [60]. It should be noted, however, that migalastat is only indicated in patients with an amenable *GLA* variant [13]. High healthcare resource utilization was apparent across all studies [40, 61, 63, 64, 66, 69, 70], with higher resource use among patients presenting with renal complications than in those with uncomplicated disease [66].

The cost of ERT should be considered in the context of the impact of treatment status on QoL. Data from registry studies have shown that treatment with ERT attenuates disease progression and reduces the risk of cardiovascular and renal diseases [83]. For instance, Hughes and colleagues demonstrated that prompt treatment with ERT reduces the risk of cardiovascular and renal events in both men and women with FD, and in classical and non-classical disease [84]. Moreover, treatment of younger patients with ERT may be more beneficial than delaying treatment into later adulthood. A study by Parini and colleagues found that, in patients aged under 18 years or 18–30 years who were treated with ERT, renal and cardiac functional decline was attenuated compared with patients who began ERT after the age of 30 [85]. In pediatric patients, ERT was effective in reducing the FD symptoms of pain in girls and gastrointestinal distress in boys, while maintaining stable cardiac and renal parameters [86]. Therefore, the delayed disease progression associated with ERT is likely to benefit patients’ QoL and may also offset some of the healthcare resource use and costs associated with the consequences of disease progression and disease complications, such as hospital visits.

Notwithstanding the clinical benefits of treatment and the expected improvement in patients’ QoL, certain limitations of treatment may also exert an effect on QoL. Limitations of ERT may include the inconvenience of lifelong intravenous infusions, the potential of adverse reactions (fever, chills) in response to infusions, and a potential loss of efficacy due to the production of antidrug neutralizing antibodies. For migalastat, limitations may include the fact that therapy is only an option for patients with amenable *GLA* mutations, as well as the potential occurrence of adverse events, such as headache [87]. Indeed, Lloyd and colleagues showed that such limitations of treatment are associated with health disutilities, which may influence patient treatment choices to an extent [72]. Consensus statements on the management of FD suggest that therapy-related burdens impacting QoL should be addressed by physicians if possible [74, 88], but studies are needed to further understand the association between the burden of current and emerging FD therapies on patients’ QoL, and these will be critical to better inform disease management.

Few systematic reviews reporting on the burden of FD have been published; here, we provide a comprehensive review, capturing several additional years of studies and combining the humanistic and economic evidence on the overall burden of FD. A systematic review of ERT in FD by Connock and colleagues in 2006 found insufficient data on health utility or economic evaluations to draw robust conclusions on the cost-effectiveness of ERT, likely due to the more limited evidence available at the time of reporting [89]. Similar to the present review, a systematic review by Arends and colleagues in 2015 reported reduced QoL in patients with FD compared with the general population, with renal disease, pain, and age all as influencing factors [4]. However, the economic burden of FD was not reported. A systematic review including an economic evaluation of ERT in FD concluded that FD carries a substantial cost burden, the majority of which can be attributed to treatment with ERT [90], in line with the findings of the present study. However, that review did not explore the humanistic burden of disease.

Although this comprehensive systematic review covered a wide range of QoL tools and various aspects of the economic and resource burden of FD, the findings are subject to some limitations. First, the heterogeneity in populations and measures used across studies, including differences in treatment status, limits the ability to make direct comparisons or to combine results. Second, we did not identify any studies that evaluated the impact of oral chaperone therapy on QoL. Moreover, there are limited data regarding the economic and resource burden of oral therapies. The included studies generally lacked comparison with other disease cohorts—for example, patients with cancer, CKD, heart failure, or type 2 diabetes—and, instead, favored comparisons with aged-matched healthy populations. This limits our ability to interpret these findings on FD in context with other clinical populations. Finally, inherent limitations of SLRs include potential publication bias and potential selection bias within the studies included in the review, particularly in relation to patient recruitment and outcome reporting. The quality of the economic studies included in this SLR was assessed using the CHEERS, Philips, and NICE single technology appraisal-adapted Drummond’s checklists. The included studies met most checklist criteria, suggesting that quality issues of the included studies and potential selection bias within the studies had minimal impact on the interpretation of the SLR results. An additional limitation of the present review is that the quality of the publications on the humanistic burden of disease was not also determined.

In conclusion, there remains a substantial disease burden in patients with FD, indicating an unmet management need. Closer monitoring of QoL with disease-specific instruments and a greater focus on QoL in patient management, as well as increased awareness and adoption of consensus recommendations, may help to address this unmet need. Disease-specific QoL instruments may improve the ability to measure the impact of FD and may provide more specific information on the effect of treatments on different disease phenotypes. The inclusion of key symptoms, such as fatigue, as clinical trial endpoints will also help to establish the impact of treatment on the burden of disease. Furthermore, increased efforts are required to reduce the high healthcare costs associated with FD, which may include utilizing community-based resources as an alternative to hospital visits. As suggested both by Milligan and colleagues and by Beck and colleagues, at-home infusions and self-administration may help to alleviate the burden associated with ERT [91, 92]. Overall, integrating information from QoL and economic assessments may help to identify interventions that are likely to be of most value for specific patient populations, in terms of impact on patients’ QoL and on cost to payers. This could potentially enable better targeting and earlier initiation of treatment, where appropriate, leading to a positive impact on cost-effectiveness in the management of FD.

### Supplementary Information

**Additional file 1.** Supplementary Tables.

## Data Availability

The data included in this report are from the published literature; all articles meeting the search criteria are listed and full publication details are provided.

## Abbreviations

α-Gal A: α-Galactosidase A
ABCL: Adult behavior checklist
AFQOL: Adult Fabry Quality of Life Scale
ASEBA: Achenbach System of Empirically Based Assessment
ASR: Adult self-report
BASC: Behavior Assessment Scale for Children
BDI-II: Beck Depression Inventory-II
BPI: Brief Pain Inventory
BPI-SF3: Brief Pain Inventory – Short-Form item 3
CDI: Children’s Depression Inventory
CES-D: Centre of Epidemiologic Studies Depression Scale
CHEERS: Consolidated Health Economic Evaluation Reporting Standards
CHQ: Child Health Questionnaire
CI: Confidence interval
CKD: Chronic kidney disease
EED: Economic Evaluations Database
eGFR: Estimated glomerular filtration rate
EMA: European Medicines Agency
EQ-5D: EuroQol five dimension
ERT: Enzyme replacement therapy
ESRD: End-stage renal disease
ESS: Epworth Sleepiness Scale
FABPRO-GI: Fabry Disease Patient-Reported Outcome – Gastrointestinal
FD: Fabry disease
FDA: Food and Drug Administration
FD-PRO: Fabry Disease – Patient-Reported Outcome
FPHPQ: Fabry-specific Pediatric Health and Pain Questionnaire
FSS: Fatigue Severity Scale
Gb3: Globotriaosylceramide
GL-3: Globotriaosylceramide
*GLA*: α-Galactosidase A gene
HADS: Hospital Anxiety and Depression Scale
HAM-D: Hamilton Rating Scale for Depression
HTA: Health technology assessment
ICER: Incremental cost-effectiveness ratio
IQR: Interquartile range
ISPOR: Professional Society for Health Economics and Outcomes Research
JPY: Japanese yen
MMSE: Mini Mental State Examination
MSSI: Mainz Severity Score Index
NHS: National Health Service
NICE: National Institute for Health and Care Excellence
NIHR: National Institute for Health Research
NRS: Numerical Rating Scale
NUCOG: Neuropsychiatric Unit Cognitive Screen
PedsQL: Pediatric Quality of Life Inventory
PRISMA: Preferred Reporting Items for Systematic Reviews and Meta-Analysis
PSS-4: Perceived Stress Scale – 4 items
QALY: Quality-adjusted life-year
QoL: Quality of life
RBD: Rapid eye movement sleep behavior disorder
RBDSQ: RBD Screening Questionnaire
RRT: Renal replacement therapy
SD: Standard deviation
SF-12: 12-Item Short-Form Health Survey
SF-36: 36-Item Short-Form Health Survey
SGA: Subgroup analysis
SLR: Systematic literature review
SSIEM: Society for the Study of Inborn Errors of Metabolism
SSN: Servizio Sanitario Nazionale
VAS: Visual analog scale
WHO: World Health Organization
WHO QoL-100: 100-item World Health Organization Quality of Life scale
WORLD: We’re Organizing Research on Lysosomal Diseases
YFEOD: Year free of end-organ damage
