# Supplementary material for: A systematic literature review on the health-related quality of life and economic burden of Fabry disease
Source: Orphanet J Rare Dis. 2024 Apr 30;19:181. doi: 10.1186/s13023-024-03131-y (PMC11062018; doi:10.1186/s13023-024-03131-y)
Supplement: Supplementary file 1 — Additional file 1. Supplementary Tables. [file 13023_2024_3131_MOESM1_ESM.docx]

**A systematic literature review on the health-related quality of life and economic burden of Fabry disease**

**SUPPLEMENTARY INFORMATION**

**Authors**

Ana Jovanovic^1^, Eve Miller-Hodges^2^, Felicia Castriota^3^, Shweta Takyar^4^, Heena Howitt^5^, Olulade Ayodele^3^

**Affiliations**

^1^ The Mark Holland Metabolic Unit, Northern Care Alliance NHS Foundation Trust, Salford, UK

^2^ Centre for Cardiovascular Science, Queen's Medical Research Institute, University of Edinburgh, Edinburgh, UK

^3^ Takeda Development Center Americas, Cambridge, MA, USA

^4^ Parexel, Mohali, India

^5^ Takeda UK Ltd, London, UK

**SUPPLEMENTARY INFORMATION**

**Supplementary Table 1.** Summary of search strategy and hits for humanistic (A) and economic (B) searches

**A**

| **Embase®** | | Search hits |
| --- | --- | --- |
| **Disease facet (Fabry disease)** | | |
| 1 | ‘Fabry Disease’/syn | 8982 |
| 2 | fabry*:ab,ti,kw | 9419 |
| 3 | ‘angiokeratoma’ NEAR/3 ‘diffusum’ OR ‘angiokeratoma diffuse’ | 297 |
| 4 | ‘Diffuse angiokeratoma*’ OR ‘angiokeratoma corporis diffusum’ | 305 |
| 5 | ‘anderson-fabry disease’ OR ‘anderson fabry disease’ OR ‘anderson fabry’ OR ‘fabrys disease’ OR ‘anderson fabrys disease’ OR ‘afd’:ab,ti | 1290 |
| 6 | ‘Galactosidase’ NEAR/3 ‘deficiency’ | 1145 |
| 7 | ‘alpha galactosidase’/syn OR ‘α-galactosidase’ | 6499 |
| 8 | (‘alpha galactosidase’ OR ‘alpha-galactosidase’ OR ‘α-galactosidase’):ab,ti,kw | 4522 |
| 9 | ‘GLA’ NEAR/3 ‘deficiency’ OR ‘α-galactosidase A deficiency’ OR ‘alpha galactosidase A deficiency disease’ OR ‘α-galactosidase A deficiency disease’ OR ‘alpha-galactosidase A deficiency disease’ OR ‘alpha galactosidase A deficiency’ | 266 |
| 10 | ‘Ceramide Trihexosidase Deficiency’ OR ‘Glycolipid lipidosis’ OR ‘Hereditary Dystopic Lipidosis’ | 8 |
| 11 | #1 OR #2 OR #3 OR #4 OR #5 OR #6 OR #7 OR #8 OR #9 OR #10 | 14,980 |
| **Study design facet (quality of life)** | | |
| 12 | ‘quality of life’/syn OR ‘health related quality of life’/exp OR ‘health-related quality of life’/exp | 696,702 |
| 13 | ‘HRQoL’/exp OR ‘HRQL’/exp OR ‘QoL’/exp OR ‘Quality life’ OR ‘Life quality’ | 583,276 |
| 14 | ‘quality adjusted life’ OR ‘quality-adjust-life’ OR ‘qaly’ OR ‘qald’ OR ‘qale’ OR ‘qtime’ OR ‘quality adjusted life year’/syn | 36,788 |
| 15 | ‘quality’ NEAR/3 ‘life’ | 703,354 |
| 16 | ‘QOLS’ OR ‘Quality of life scale’ | 5151 |
| 17 | ‘sf6D’ OR ‘sf 6D’ OR ‘sf-6D’ OR ‘short form 6D’ OR ‘shortform 6D’ OR ‘shortform6D’ | 1728 |
| 18 | ‘sf6’ OR ‘sf 6’ OR ‘sf-6’ OR ‘short form 6’ OR ‘shortform 6’ OR ‘shortform6’ OR ‘sf six’ OR ‘shortform six’ OR ‘short form six’ OR ‘Short-Form 6-Dimensions’ OR ‘Short-Form 6’ | 2834 |
| 19 | ‘sf8’ OR ‘sf 8’ OR ‘sf-8’ OR ‘shortform 8’ OR ‘shortform8’ OR ‘sf eight’ OR ‘shortform eight’ OR ‘short form eight’ OR ‘Short-Form 8-Dimensions’ OR ‘Short-Form 8’ OR ‘short form 8’/syn | 1290 |
| 20 | ‘sf12’ OR ‘sf 12’ OR ‘sf-12’ OR ‘shortform 12’ OR ‘shortform12’ OR ‘sf twelve’ OR ‘shortform twelve’ OR ‘short form twelve’ OR ‘short form 12’/syn | 13,010 |
| 21 | ‘sf20’ OR ‘sf 20’ OR ‘sf-20’ OR ‘shortform 20’ OR ‘shortform20’ OR ‘sf twenty’ OR ‘shortform twenty’ OR ‘short form twenty’ OR ‘short form 20’/syn | 567 |
| 22 | ‘sf36’ OR ‘sf 36’ OR ‘sf-36’ OR ‘shortform 36’ OR ‘shortform36’ OR ‘sf thirtysix’ OR ‘sf thirty six’ OR ‘shortform thirtysix’ OR ‘shortform thirty six’ OR ‘short form thirtysix’ OR ‘short form thirty six’ OR ‘short form 36’/syn | 62,191 |
| 23 | ‘euroqol’ OR ‘euro-qol’ OR ‘euro qol’ OR ‘eq5d’ OR ‘eq-5d’ OR ‘eq 5d’ OR ‘European Quality of Life 5 Dimensions questionnaire’/syn | 27,201 |
| 24 | ‘European Quality of Life 5 Dimensions 3 Level questionnaire’/syn | 2798 |
| 25 | ‘European Quality of Life 5 Dimensions 5 Level questionnaire’/syn | 4254 |
| 26 | ‘European Quality of Life 5 Dimensions Visual Analogue Scale’/syn | 3465 |
| 27 | ‘RAND 36’ OR ‘RAND36’ OR ‘RAND-36’ OR (‘Research’ NEAR/2 ‘Development 36’) OR ‘RAND-36 item Health Survey’ OR ‘RAND 36 item Health Survey’ | 1439 |
| 28 | ‘Pediatric Quality of Life Inventory’/syn OR ‘PedsQL’ | 4793 |
| 29 | ‘Fabry-specific pediatric health and pain questionnaire’ OR ‘FPHPQ’ | 4 |
| 30 | (‘AFD specific questionnaire’) OR (Fabry* AND ‘specific questionnaire’) OR (‘AFD specific questions’) OR (Fabry* AND ‘specific questions’) | 9 |
| 31 | ‘Disease severity scoring system’ OR ‘DS3’ | 263 |
| 32 | ‘pain assessment’/syn | 191,820 |
| 33 | ‘BPI’:ab,ti OR ‘Brief pain inventory’/exp | 7447 |
| 34 | ‘McGill Pain Questionnaire’/syn OR ‘McGill pain’ | 4280 |
| 35 | ‘FPQ’ OR ‘Fabry pain questionnaire’ | 145 |
| 36 | ‘Fabry disease-specific questionnaire’ OR ‘FD-specific questionnaire’ | 2 |
| 37 | ‘EQ-VAS’ OR ‘EQ VAS’ OR ‘EuroQol Visual Analog Scale’ OR ‘visual analog scale’ OR (‘visual’ NEXT/1 analog* AND analog* NEXT/1 scale*) | 126,693 |
| 38 | #12 OR #13 OR #14 OR #15 OR #16 OR #17 OR #18 OR #19 OR #20 OR #21 OR #22 OR #23 OR #24 OR #25 OR #26 OR #27 OR #28 OR #29 OR #30 OR #31 OR #32 OR #33 OR #34 OR #35 OR #36 OR #37 | 909,021 |
| **Study design facet (Health state utility)** | | |
| 39 | ‘utility’:ab,ti OR utilit*:ab,ti OR (‘health’ NEAR/2 utilit*):ab,ti OR ‘health state utility’ OR ‘HSUV’ OR ‘health state utility value’ OR ‘health state utility values’ OR ‘HSUVs’ | 328,688 |
| 40 | ‘health’ AND (‘state’ NEXT/1 utilit*) OR ‘utility score’ OR ‘health utility’ | 4946 |
| 41 | (‘health’ NEXT/1 state* AND state* NEXT/1 preference*) | 282 |
| 42 | health*year*equivalent:ab,ti OR ‘hye’:ab,ti OR ‘hyes’:ab,ti | 154 |
| 43 | ‘health utility index’:ab,ti OR ‘hui’:ab,ti OR ‘hui1’:ab,ti OR ‘hui2’:ab,ti OR ‘hui3’:ab,ti | 2890 |
| 44 | (utilit* NEAR/2 (measure* OR outcome* OR state* OR health OR score* OR weight* OR ‘analysis’ OR ‘analyses’)):ab,ti | 16,467 |
| 45 | ‘Rosser’ OR ‘willingness to pay’ OR ‘willingness’ NEAR/2 ‘pay’ OR 'discrete choice' NEXT/1 experiment* | 16,409 |
| 46 | (utilit* NEXT/1 (score* OR value* OR evaluation*)) | 6414 |
| 47 | ‘standard gamble’ OR (‘standard’ NEAR/2 gamble*) | 1190 |
| 48 | ‘Time trade off’ OR ‘time tradeoff’ OR ‘time trade-off’ OR (‘time’ NEAR/2 trade*off) OR ‘TTO’ OR ‘time trade’ | 3244 |
| 49 | ‘Health status indicator’:ab,ti OR ‘activities of daily living’:ab,ti OR ‘Health survey’:ab,ti | 80,746 |
| 50 | ‘disability adjusted life’ OR ‘daly’ OR ‘disability adjusted’ OR ‘disability adjusted life year’ | 23,023 |
| 51 | disutilit* OR ‘Utility decrement’ OR ‘disutilities’ OR ‘disutility’ OR ‘disutility value’ | 1168 |
| 52 | ‘Utility instrument’ OR ‘Multi-attribute utility instrument’ OR ‘MAUI’ | 1781 |
| 53 | ‘euroqol’ OR ‘euro-qol’ OR ‘euro qol’ OR ‘eq5d’ OR ‘eq-5d’ OR ‘eq 5d’ OR ‘European Quality of Life 5 Dimensions questionnaire’/syn | 27,201 |
| 54 | ‘European Quality of Life 5 Dimensions 3 Level questionnaire’/syn | 2798 |
| 55 | ‘European Quality of Life 5 Dimensions 5 Level questionnaire’/syn | 4254 |
| 56 | ‘sf6D’ OR ‘sf 6D’ OR ‘sf-6D’ OR ‘short form 6D’ OR ‘shortform 6D’ OR ‘shortform6D’ OR ‘VR-6D’ | 1735 |
| 57 | ‘sf6’ OR ‘sf 6’ OR ‘sf-6’ OR ‘short form 6’ OR ‘shortform 6’ OR ‘shortform6’ OR ‘sf six’ OR ‘shortform six’ OR ‘short form six’ OR ‘Short-Form 6-Dimensions’ OR ‘Short-Form 6’ | 2834 |
| 58 | ‘sf36’ OR ‘sf 36’ OR ‘sf-36’ OR ‘shortform 36’ OR ‘shortform36’ OR ‘sf thirtysix’ OR ‘sf thirty six’ OR ‘shortform thirtysix’ OR ‘shortform thirty six’ OR ‘short form thirtysix’ OR ‘short form thirty six’ OR ‘short form 36’/syn | 62,191 |
| 59 | ‘Quality of Wellbeing’ OR ‘QWB’ | 454 |
| 60 | ‘AQoL’ OR ‘Assessment quality of life’ | 638 |
| 61 | ‘quality adjusted life’ OR ‘quality-adjust-life’ OR ‘qaly’ OR ‘qald’ OR ‘qale’ OR ‘qtime’ OR ‘quality adjusted life year’/syn | 36,788 |
| 62 | ((preference* OR utilit*) AND ‘quality of life’):ab,ti,kw | 22,552 |
| 63 | (Preference* OR Utilit*) NEAR/2 (elicit* OR population* OR measure*) | 7693 |
| 64 | ‘weight’ NEAR/3 ‘preference’ | 381 |
| 65 | ‘qtwist’ OR ‘q twist’ | 260 |
| 66 | ‘life expectancy’/de AND ‘disability’/de | 1364 |
| 67 | Disabilit* NEAR/3 adjust* NEAR/3 (life-year* OR life-expect* OR lifeyear*) | 6166 |
| 68 | Disabilit* NEAR/6 ‘life’ NEXT/1 (year* OR expect*) | 6904 |
| 69 | #39 OR #40 OR #41 OR #42 OR #43 OR #44 OR #45 OR #46 OR #47 OR #48 OR #49 OR #50 OR #51 OR #52 OR #53 OR #54 OR #55 OR #56 OR #57 OR #58 OR #59 OR #60 OR #61 OR #62 OR #63 OR #64 OR #65 OR #66 OR #67 OR #68 | 542,578 |
| **Combined facet** | | |
| 70 | #38 OR #69 | 1,297,727 |
| 71 | #11 AND #70 | 728 |
| 72 | #71 AND [animals]/lim NOT ([humans]/lim AND [animals]/lim) | 17 |
| 73 | #71 AND ([conference review]/lim OR [editorial]/lim OR [letter]/lim OR [note]/lim OR [review]/lim OR [short survey]/lim) | 176 |
| 74 | #72 OR #73 | 192 |
| 75 | #71 NOT #74 | 536 |
| 76 | #71 NOT #74 AND [english]/lim (hits as on date January 27, 2020) | 414 |
| 77 | #71 NOT #74 AND [english]/lim (Data inception to January 19, 2022) | 520 |

| **Cochrane** | | **Search hits** |
| --- | --- | --- |
| **Disease facet (Fabry disease)** | | |
| 1 | MeSH descriptor: [Fabry Disease] explode all trees | 75 |
| 2 | fabry*:ti,ab,kw | 245 |
| 3 | “angiokeratoma” NEAR/3 “diffusum” OR “angiokeratoma diffuse” | 1 |
| 4 | “Diffuse angiokeratoma*” OR “angiokeratoma corporis diffusum” | 1 |
| 5 | “anderson fabry disease” OR “anderson fabry” OR “fabrys disease” OR “anderson fabrys disease” OR “afd”:ti,ab,kw | 30 |
| 6 | “Galactosidase” NEAR/3 “deficiency” | 27 |
| 7 | MeSH descriptor: [alpha-galactosidase] explode all trees | 50 |
| 8 | “α-galactosidase” | 218 |
| 9 | (“alpha galactosidase” OR “alpha-galactosidase” OR “α-galactosidase”):ti,ab,kw | 114 |
| 10 | “GLA” NEAR/3 “deficiency” OR “alpha galactosidase A deficiency disease” OR “alpha galactosidase A deficiency” | 4 |
| 11 | “Ceramide Trihexosidase Deficiency” OR “Glycolipid lipidosis” OR “Hereditary Dystopic Lipidosis” | 0 |
| 12 | #1 OR #2 OR #3 OR #4 OR #5 OR #6 OR #7 OR #8 OR #9 OR #10 OR #11 | 511 |
| **Study design facet (Quality of life)** | | |
| 13 | MeSH descriptor: [quality of life] explode all trees | 27,298 |
| 14 | “health related quality of life” | 20,409 |
| 15 | “HRQoL” OR “HRQL” OR “QoL” OR “Quality life” OR “Life quality” | 35,150 |
| 16 | MeSH descriptor: [Quality-Adjusted Life Years] explode all trees | 1409 |
| 17 | “quality adjusted life” OR “quality-adjust-life” OR “qaly” OR “qald” OR “qale” OR “qtime” | 6129 |
| 18 | “quality” NEAR/3 “life” | 133,772 |
| 19 | “QOLS” OR “Quality of life scale” | 1979 |
| 20 | “sf6D” OR “sf 6D” OR “sf-6D” OR “short form 6D” OR “shortform 6D” OR “shortform6D” | 361 |
| 21 | “sf6” OR “sf 6” OR “sf-6” OR “short form 6” OR “shortform 6” OR “shortform6” OR “sf six” OR “shortform six” OR “short form six” | 291 |
| 22 | “sf8” OR “sf 8” OR “sf-8” OR “shortform 8” OR “shortform8” OR “sf eight” OR “shortform eight” OR “short form eight” OR “short form 8” | 320 |
| 23 | “sf12” OR “sf 12” OR “sf-12” OR “shortform 12” OR “shortform12” OR “sf twelve” OR “shortform twelve” OR “short form twelve” OR “short form 12” | 3453 |
| 24 | “sf20” OR “sf 20” OR “sf-20” OR “shortform 20” OR “shortform20” OR “sf twenty” OR “shortform twenty” OR “short form twenty” OR “short form 20” | 114 |
| 25 | “sf36” OR “sf 36” OR “sf-36” OR “shortform 36” OR “shortform36” OR “sf thirtysix” OR “sf thirty six” OR “shortform thirtysix” OR “shortform thirty six” OR “short form thirtysix” OR “short form thirty six” OR “short form 36” | 16,484 |
| 26 | “euroqol” OR “euro-qol” OR “euro qol” OR “eq5d” OR “eq-5d” OR “eq 5d” OR “European Quality of Life 5 Dimensions questionnaire” | 11,572 |
| 27 | “European Quality of Life 5 Dimensions 3 Level questionnaire” | 238 |
| 28 | “European Quality of Life 5 Dimensions 5 Level questionnaire” | 497 |
| 29 | “European Quality of Life 5 Dimensions Visual Analogue Scale” | 163 |
| 30 | “RAND 36” OR “RAND36” OR “RAND-36” OR (“Research” NEAR/2 “Development 36”) OR “RAND 36 item Health Survey” | 422 |
| 31 | “Pediatric Quality of Life Inventory” OR “PedsQL” | 798 |
| 32 | (“AFD specific questionnaire”) OR (Fabry* AND “specific questionnaire”) OR (“AFD specific questions”) OR (Fabry* AND “specific questions”) | 0 |
| 33 | “Disease severity scoring system” OR “DS3” | 14 |
| 34 | MeSH descriptor: [pain assessment] explode all trees | 22,463 |
| 35 | “BPI”:ti,ab,kw OR “Brief pain inventory” | 2334 |
| 36 | “McGill Pain Questionnaire” OR “McGill pain” | 1893 |
| 37 | “FPQ” OR “Fabry pain questionnaire” | 13 |
| 38 | “Fabry disease-specific questionnaire” | 0 |
| 39 | “EQ-VAS” OR “EQ VAS” OR “EuroQol Visual Analog Scale” OR “visual analog scale” OR (“visual” NEXT/1 analog* AND analog* NEXT/1 scale*) | 54,661 |
| 40 | #13 OR #14 OR #15 OR #16 OR #17 OR #18 OR #19 OR #20 OR #21 OR #22 OR #23 OR #24 OR #25 OR #26 OR #27 OR #28 OR #29 OR #30 OR #31 OR #32 OR #33 OR #34 OR #35 OR #36 OR #37 OR #38 OR #39 | 202,899 |
| **Combined facet** | | |
| 41 | #12 AND #40 | 214 |
| 42 | #12 AND #23 in Cochrane Reviews, Cochrane Protocols, Trials and Clinical Answers (Word variations have been searched) (search hits as on date 27^th^ January 2020) | 171 |
| 43 | #12 AND #23 in Cochrane Reviews, Cochrane Protocols, Trials and Clinical Answers (Word variations have been searched) (Data inception to January 19, 2022) | 215 |

| **PubMed (MEDLINE® and MEDLINE® In-Process)** | | **Search hits** |
| --- | --- | --- |
| **Disease facet (Fabry disease)** | | |
| 1 | “Fabry Disease” | 4700 |
| 2 | fabry*[Title/Abstract] | 8737 |
| 3 | “angiokeratoma” AND “diffusum” OR “angiokeratoma diffuse” | 265 |
| 4 | “Diffuse angiokeratoma*” OR “angiokeratoma corporis diffusum” | 274 |
| 5 | “anderson-fabry disease” OR “anderson fabry disease” OR “anderson fabry” OR “fabrys disease” OR “anderson fabrys disease” OR “afd”[Title/Abstract] | 963 |
| 6 | “Galactosidase” AND “deficiency” | 2571 |
| 7 | “alpha galactosidase” OR “α-galactosidase” | 4368 |
| 8 | (“alpha galactosidase” OR “alpha-galactosidase” OR “α-galactosidase”)[Title/Abstract] | 4368 |
| 9 | “GLA” AND “deficiency” OR “α-galactosidase A deficiency” OR “alpha galactosidase A deficiency disease” OR “α-galactosidase A deficiency disease” OR “alpha-galactosidase A deficiency disease” OR “alpha galactosidase A deficiency” | 5784 |
| 10 | “Ceramide Trihexosidase Deficiency” OR “Glycolipid lipidosis” OR “Hereditary Dystopic Lipidosis” | 8 |
| 11 | #1 OR #2 OR #3 OR #4 OR #5 OR #6 OR #7 OR #8 OR #9 OR #10 | 13,910 |
| **Study design facet (Quality of life)** | | |
| 12 | “quality of life” OR “health related quality of life” OR “health-related quality of life” | 384,290 |
| 13 | “HRQoL” OR “HRQL” OR “QoL” OR “Quality life” OR “Life quality” | 75,744 |
| 14 | “quality adjusted life” OR “quality-adjust-life” OR “qaly” OR “qald” OR “qale” OR “qtime” OR “quality adjusted life year” | 22,299 |
| 15 | “quality” AND “life” | 467,360 |
| 16 | “QOLS” OR “Quality of life scale” | 3037 |
| 17 | “sf6D” OR “sf 6D” OR “sf-6D” OR “short form 6D” OR “shortform 6D” OR “shortform6D” | 940 |
| 18 | “sf6” OR “sf 6” OR “sf-6” OR “short form 6” OR “shortform 6” OR “shortform6” OR “sf six” OR “shortform six” OR “short form six” OR “Short-Form 6-Dimensions” OR “Short-Form 6” | 6589 |
| 19 | “sf8” OR “sf 8” OR “sf-8” OR “shortform 8” OR “shortform8” OR “sf eight” OR “shortform eight” OR “short form eight” OR “Short-Form 8-Dimensions” OR “Short-Form 8” OR “short form 8” | 9343 |
| 20 | “sf12” OR “sf 12” OR “sf-12” OR “shortform 12” OR “shortform12” OR “sf twelve” OR “shortform twelve” OR “short form twelve” OR “short form 12” | 7797 |
| 21 | “sf20” OR “sf 20” OR “sf-20” OR “shortform 20” OR “shortform20” OR “sf twenty” OR “shortform twenty” OR “short form twenty” OR “short form 20” | 5443 |
| 22 | “sf36” OR “sf 36” OR “sf-36” OR “shortform 36” OR “shortform36” OR “sf thirtysix” OR “sf thirty six” OR “shortform thirtysix” OR “shortform thirty six” OR “short form thirtysix” OR “short form thirty six” OR “short form 36” | 28,651 |
| 23 | “euroqol” OR “euro-qol” OR “euro qol” OR “eq5d” OR “eq-5d” OR “eq 5d” OR “European Quality of Life 5 Dimensions questionnaire” | 13,883 |
| 24 | “European Quality of Life 5 Dimensions 3 Level questionnaire” | 2 |
| 25 | “European Quality of Life 5 Dimensions 5 Level questionnaire” | 2 |
| 26 | “European Quality of Life 5 Dimensions Visual Analogue Scale” | 5 |
| 27 | “RAND 36” OR “RAND36” OR “RAND-36” OR (“Research” AND “Development 36”) OR “RAND-36 item Health Survey” OR “RAND 36 item Health Survey” | 1042 |
| 28 | “Pediatric Quality of Life Inventory” OR “PedsQL” | 2453 |
| 29 | “Fabry-specific pediatric health and pain questionnaire” OR “FPHPQ” | 2 |
| 30 | (“AFD specific questionnaire”) OR (Fabry* AND “specific questionnaire”) OR (“AFD specific questions”) OR (Fabry* AND “specific questions”) | 153 |
| 31 | “Disease severity scoring system” OR “DS3” | 157 |
| 32 | “pain assessment” | 6424 |
| 33 | “BPI”[Title/Abstract] OR “Brief pain inventory” | 4302 |
| 34 | “McGill Pain Questionnaire” OR “McGill pain” | 2344 |
| 35 | “FPQ” OR “Fabry pain questionnaire” | 110 |
| 36 | “Fabry disease-specific questionnaire” OR “FD-specific questionnaire” | 23 |
| 37 | “EQ-VAS” OR “EQ VAS” OR “EuroQol Visual Analog Scale” OR “visual analog scale” OR (“visual” AND analog* AND analog* AND scale*) | 69,390 |
| 38 | #12 OR #13 OR #14 OR #15 OR #16 OR #17 OR #18 OR #19 OR #20 OR #21 OR #22 OR #23 OR #24 OR #25 OR #26 OR #27 OR #28 OR #29 OR #30 OR #31 OR #32 OR #33 OR #34 OR #35 OR #36 OR #37 | 559,621 |
| **Study design facet (Health state utility)** | | |
| 39 | “utility”[Title/Abstract] OR utilit*[Title/Abstract] OR (“health” AND utilit*)[Title/Abstract] OR “health state utility” OR “HSUV” OR “health state utility value” OR “health state utility values” OR “HSUVs” | 237,944 |
| 40 | “health” AND (“state” AND utilit*) OR “utility score” OR “health utility” | 10,529 |
| 41 | (“health” AND state* AND state* AND preference*) | 23,259 |
| 42 | health*year*equivalent[Title/Abstract] OR “hye”[Title/Abstract] OR “hyes”[Title/Abstract] | 85 |
| 43 | “health utility index”[Title/Abstract] OR “hui”[Title/Abstract] OR “hui1”[Title/Abstract] OR “hui2”[Title/Abstract] OR “hui3”[Title/Abstract] | 1916 |
| 44 | (utilit* AND (measure* OR outcome* OR state* OR health OR score* OR weight* OR “analysis” OR “analyses”))[Title/Abstract] | 187,437 |
| 45 | “Rosser” OR “willingness to pay” OR “willingness” AND “pay” OR “discrete choice” AND experiment* | 2744 |
| 46 | (utilit* AND (score* OR value* OR evaluation*)) | 87,458 |
| 47 | “standard gamble” OR (“standard” AND gamble*) | 1485 |
| 48 | “Time trade off” OR “time tradeoff” OR “time trade-off” OR (“time” AND trade*off) OR “TTO” OR “time trade” | 2224 |
| 49 | “Health status indicator”[Title/Abstract] OR “activities of daily living”[Title/Abstract] OR “Health survey”[Title/Abstract] | 64,258 |
| 50 | “disability adjusted life” OR “daly” OR “disability adjusted” OR “disability adjusted life year” | 17,514 |
| 51 | disutilit* OR “Utility decrement” OR “disutilities” OR “disutility” OR “disutility value” | 599 |
| 52 | “Utility instrument” OR “Multi-attribute utility instrument” OR “MAUI” | 588 |
| 53 | “euroqol” OR “euro-qol” OR “euro qol” OR “eq5d” OR “eq-5d” OR “eq 5d” OR “European Quality of Life 5 Dimensions questionnaire” | 13,883 |
| 54 | “European Quality of Life 5 Dimensions 3 Level questionnaire” | 2 |
| 55 | “European Quality of Life 5 Dimensions 5 Level questionnaire” | 2 |
| 56 | “sf6D” OR “sf 6D” OR “sf-6D” OR “short form 6D” OR “shortform 6D” OR “shortform6D” OR “VR-6D” | 943 |
| 57 | “sf6” OR “sf 6” OR “sf-6” OR “short form 6” OR “shortform 6” OR “shortform6” OR “sf six” OR “shortform six” OR “short form six” OR “Short-Form 6-Dimensions” OR “Short-Form 6” | 6589 |
| 58 | “sf36” OR “sf 36” OR “sf-36” OR “shortform 36” OR “shortform36” OR “sf thirtysix” OR “sf thirty six” OR “shortform thirtysix” OR “shortform thirty six” OR “short form thirtysix” OR “short form thirty six” OR “short form 36” | 28,651 |
| 59 | “Quality of Wellbeing” OR “QWB” | 216 |
| 60 | “AQoL” OR “Assessment quality of life” | 272 |
| 61 | “quality adjusted life” OR “quality-adjust-life” OR “qaly” OR “qald” OR “qale” OR “qtime” OR “quality adjusted life year” | 22,299 |
| 62 | ((preference* OR utilit*) AND “quality of life”)[Title/Abstract] | 16,617 |
| 63 | (Preference* OR Utilit*) AND (elicit* OR population* OR measure*) | 133,217 |
| 64 | “weight” AND “preference” | 5894 |
| 65 | “qtwist” OR “q twist” | 123 |
| 66 | “life expectancy” AND “disability” | 2268 |
| 67 | Disabilit* AND adjust* AND (life-year* OR life-expect* OR lifeyear*) | 5101 |
| 68 | Disabilit* AND “life” AND (year* OR expect*) | 26,963 |
| 69 | #39 OR #40 OR #41 OR #42 OR #43 OR #44 OR #45 OR #46 OR #47 OR #48 OR #49 OR #50 OR #51 OR #52 OR #53 OR #54 OR #55 OR #56 OR #57 OR #58 OR #59 OR #60 OR #61 OR #62 OR #63 OR #64 OR #65 OR #66 OR #67 OR #68 | 456,667 |
| **Combined facet** | | |
| 70 | #38 OR #69 | 915,775 |
| 71 | #11 AND #70 | 420 |
| 72 | #71 AND (in process[sb] OR pubstatusaheadofprint)) (search hits as on date 27^th^ January 2020) | 4 |
| 73 | #71 AND (in process[sb] OR pubstatusaheadofprint)) (Data inception to January 19, 2022) | 11 |

**B**

| **EMBASE** | | **Search hits** |
| --- | --- | --- |
| **Disease facet (Fabry disease)** | | |
| 1 | ‘Fabry Disease’/syn | 8982 |
| 2 | fabry*:ab,ti,kw | 9419 |
| 3 | ‘angiokeratoma’ NEAR/3 ‘diffusum’ OR ‘angiokeratoma diffuse’ | 297 |
| 4 | ‘Diffuse angiokeratoma*’ OR ‘angiokeratoma corporis diffusum’ | 305 |
| 5 | ‘anderson-fabry disease’ OR ‘anderson fabry disease’ OR ‘anderson fabry’ OR ‘fabrys disease’ OR ‘anderson fabrys disease’ OR ‘afd’:ab,ti | 1290 |
| 6 | ‘Galactosidase’ NEAR/3 ‘deficiency’ | 1145 |
| 7 | ‘alpha galactosidase’/syn OR ‘α-galactosidase’ | 6499 |
| 8 | (‘alpha galactosidase’ OR ‘alpha-galactosidase’ OR ‘α-galactosidase’):ab,ti,kw | 4522 |
| 9 | ‘GLA’ NEAR/3 ‘deficiency’ OR ‘α-galactosidase A deficiency’ OR ‘alpha galactosidase A deficiency disease’ OR ‘α-galactosidase A deficiency disease’ OR ‘alpha-galactosidase A deficiency disease’ OR ‘alpha galactosidase A deficiency’ | 266 |
| 10 | ‘Ceramide Trihexosidase Deficiency’ OR ‘Glycolipid lipidosis’ OR ‘Hereditary Dystopic Lipidosis’ | 8 |
| 11 | #1 OR #2 OR #3 OR #4 OR #5 OR #6 OR #7 OR #8 OR #9 OR #10 | 14,980 |
| 12 | ‘Cost’ NEAR/2 (effective* OR efficien* OR utilit* OR minimi* OR consequen* OR benefit* OR unit* OR estimate* OR variable*) | 385,766 |
| 13 | ‘budget impact analysis’ OR ‘budget impact model’ OR (‘budget impact’ NEAR/3 (method* OR analys* OR model* OR simulation* OR assessment*)) OR ‘BIA’:ab,ti,kw | 9741 |
| 14 | ‘economic evaluation’/syn OR ‘economic model’/syn OR ‘pharmacoeconomics’/syn | 538,664 |
| 15 | ‘markov’ NEAR/3 (method* OR analys* OR model* OR simulation* OR assessment* OR chain*) OR ‘hidden markov model’/syn OR ‘Markov chain’/syn | 35,295 |
| 16 | ‘monte carlo method’/syn OR (‘monte carlo’ NEAR/3 (method* OR analys* OR model* OR simulation* OR assessment* OR chain*)) OR ‘Monte Carlo simulation’ OR ‘Monte Carlo technique’ | 58,456 |
| 17 | ‘cost effectiveness analysis’/syn OR ((‘cost effectiveness’ OR ‘cost effective’) NEAR/3 (method* OR analys* OR model* OR simulation* OR assessment*)) OR ‘cost effectiveness ratio’ OR ‘cost effectiveness’ OR ‘cost-effectiveness’ OR ‘CEA’:ab,ti,kw OR ‘CER’:ab,ti,kw | 244,377 |
| 18 | ‘cost efficiency analysis’/exp OR (‘cost efficiency’ NEAR/3 (method* OR analys* OR model* OR simulation* OR assessment*)) OR ‘cost efficiency’ OR ‘cost-efficiency’ | 167,263 |
| 19 | ‘cost benefit analysis’/syn OR (‘cost benefit’ NEAR/3 (method* OR analys* OR model* OR simulation* OR assessment*)) OR ‘cost benefit’ OR ‘cost-benefit’ OR ‘cost benefit ratio’ OR ‘cost-benefit ratio’ OR ‘CBA’ OR ‘CBR’ | 126,479 |
| 20 | ‘cost utility analysis’/syn OR ((‘cost utility’ OR ‘cost utilities’) NEAR/3 (method* OR analys* OR model* OR simulation* OR assessment*)) OR ‘cost utility’ OR ‘cost-utility’ OR ‘CUA’ | 17,012 |
| 21 | ‘cost minimization analysis’/syn OR ((‘cost minimization’ OR ‘cost minimisation’) NEAR/3 (method* OR analys* OR model* OR simulation* OR assessment*)) OR ‘cost minimisation’ OR ‘cost minimization’ OR ‘cost-minimisation’ OR ‘cost-minimization’ OR 'CMA’ | 70,155 |
| 22 | ‘cost consequence analysis’/exp OR (‘cost consequence’ NEAR/3 (method* OR analys* OR model* OR simulation* OR assessment*)) OR ‘cost consequence’ OR ‘cost- consequence’ OR ‘CCA’ | 24,028 |
| 23 | ((‘cost’ OR ‘economic’) NEAR/3 (method* OR analys* OR model* OR simulation* OR assessment*)) OR ‘Health economic’ NEXT/1 stud* OR ‘decision analytic’ OR ‘decision-analytic’ | 305,632 |
| 24 | (‘decision tree’ OR ‘decision trees’):ab,ti,kw | 16,364 |
| 25 | #12 OR #13 OR #14 OR #15 OR #16 OR #17 OR #18 OR #19 OR #20 OR #21 OR #22 OR #23 OR #24 | 938,678 |
| 26 | ‘economics’/de OR ‘economic aspect’ OR ‘cost’/de OR ‘health care cost’ OR ‘drug cost’ OR ‘hospital cost’ OR ‘socioeconomics’ OR ‘health economics’ OR ‘fee’ OR ‘budget’ OR ‘hospital finance’ OR ‘financial management’ OR ‘health care financing’ OR ‘low cost’ OR ‘high cost’ OR health*care NEXT/1 cost* OR ‘health care’ NEXT/1 cost* OR ‘fiscal’ OR ‘funding’ OR ‘financial’ OR ‘finance’ OR (‘unit’ NEXT/1 cost*) OR price* OR ‘pricing’ OR Expenditure* | 1,408,892 |
| 27 | Econ* NEAR/2 (‘Burden’ OR ‘disease’ OR ‘assessment’) OR Cost* NEAR/2 (illness* OR health* OR ‘burden’ OR ‘disease’ OR ‘assessment’) | 280,552 |
| 28 | (‘Out’ NEAR/2 ‘Pocket’) OR (Patient* NEAR/2 Cost*) OR copay* OR (Privat* NEAR/2 Expendit*) OR ((Carer* OR Caregiv*) NEAR/2 (Cost* OR Expendit* OR Time)) | 48,564 |
| 29 | ‘Value’ NEAR/2 (‘Money’ OR ‘Monetary’) OR (cost* NEAR/3 (treat* OR therap*)) | 69,035 |
| 30 | ‘Costly’ OR ‘Costing’ OR pharmacoeconomic* OR pharmaco-economic* OR ‘Finances’ OR ‘Financed’ OR ‘cost analysis’ OR ‘cost assessment’ OR ‘cost study’ | 185,556 |
| 31 | ‘societal cost’ OR ‘social cost’ OR ‘social care cost’ OR ‘out of pocket’ OR ‘patient cost’ OR ‘co-payment’ OR ‘private expenditure’ OR ‘patient time’ OR ‘carer cost’ OR ‘carer expenditure’ OR ‘carer time’ OR ‘caregiver cost’ OR ‘caregiver expenditure’ OR ‘caregiver time’ OR ‘economic burden’ OR ‘cost burden’ OR ‘resource burden’ OR ‘financial burden’ OR ‘economic consequences’ OR ‘cost of illness’ OR ‘healthcare cost’ OR ‘cost of disease’ | 76,413 |
| 32 | ‘deductibles’ AND ‘coinsurance’ | 161 |
| 33 | ‘value’ NEAR/3 ‘money’ | 2767 |
| 34 | (health*care NEAR/2 (utilisation OR utilization)) OR ('health care' NEAR/2 (utilisation OR utilization)) OR (‘resource’ NEAR/2 (utilisation OR utilization OR use)) | 121,990 |
| 35 | ‘resource use’ OR ‘healthcare resources’ OR ‘resource utilization’ OR ‘resource’ OR ‘health resource’ OR ‘healthcare resource’ | 353,193 |
| 36 | ‘Patient readmission’ OR ‘Patient admission’ OR ‘Length of stay’ OR readmi* OR rehosp* OR ‘Hospital readmission’ OR ‘Reoperation’ OR ‘Emergency room’ | 455,469 |
| 37 | (‘Stay’ NEAR/2 (‘Length’ OR ‘Duration’)) | 247,467 |
| 38 | ((Outpatient* OR ‘clinic’ OR physician* OR ‘office’ OR specialist* OR ‘professional’ OR ‘practitioner’) NEAR/2 (visit* OR ‘care’)) | 188,349 |
| 39 | ((‘resource’ OR ‘staff’) NEAR/4 (utilis* OR ‘allocation’)) | 33,645 |
| 40 | ((‘high dependency’ OR ‘intensive’) NEAR/2 (‘unit’ OR ‘care’)) | 564,613 |
| 41 | ‘Resource allocation’/exp OR ‘Healthcare utilization’ | 32,852 |
| 42 | (‘healthcare’ OR ‘health care’ OR service* OR resource* OR hospital* OR ‘clinic’ OR ‘clinics’) NEAR/3 (‘visits’ OR ‘utilisation’ OR ‘utilization’ OR ‘frequency’ OR ‘number’ OR ‘access’) NEAR/3 (‘patient’ OR ‘patients’ OR ‘parents’ OR ‘subjects’ OR ‘elderly’ OR ‘adults’) | 14,777 |
| 43 | ‘health care rationing’ OR ‘medical savings accounts’ OR ‘resource allocation’ | 30,699 |
| 44 | #26 OR #27 OR #28 OR #29 OR #30 OR #31 OR #32 OR #33 OR #34 OR #35 OR #36 OR #37 OR #38 OR #39 OR #40 OR #41 OR #42 OR #43 | 2,871,484 |
| 45 | #25 OR #44 | 3,390,315 |
| 46 | #11 AND #45 | 908 |
| 47 | #46 AND [animals]/lim NOT ([humans]/lim AND [animals]/lim) | 30 |
| 48 | #46 AND ([conference review]/lim OR [editorial]/lim OR [letter]/lim OR [note]/lim OR [review]/lim OR [short survey]/lim) | 223 |
| 49 | #47 OR #48 | 252 |
| 50 | #46 NOT #49 | 656 |
| 51 | #46 NOT #49 AND [english]/lim | 631 |
| 52 | #50 AND [english]/lim AND [20-12-2019]/sd NOT [19-01-2022]/sd | 179 |

| **PubMed (MEDLINE® and MEDLINE® In-Process)** | | **Search hits** |
| --- | --- | --- |
| **Disease facet (Fabry disease)** | |  |
| 1 | “Fabry Disease” | 4700 |
| 2 | fabry*[Title/Abstract] | 8737 |
| 3 | “angiokeratoma” AND “diffusum” OR “angiokeratoma diffuse” | 265 |
| 4 | “Diffuse angiokeratoma*” OR “angiokeratoma corporis diffusum” | 274 |
| 5 | “anderson-fabry disease” OR “anderson fabry disease” OR “anderson fabry” OR “fabrys disease” OR “anderson fabrys disease” OR “afd”[Title/Abstract] | 963 |
| 6 | “Galactosidase” AND “deficiency” | 2571 |
| 7 | “alpha galactosidase” OR “α-galactosidase” | 4368 |
| 8 | (“alpha galactosidase” OR “alpha-galactosidase” OR “α-galactosidase”)[Title/Abstract] | 4368 |
| 9 | “GLA” AND “deficiency” OR “α-galactosidase A deficiency” OR “alpha galactosidase A deficiency disease” OR “α-galactosidase A deficiency disease” OR “alpha-galactosidase A deficiency disease” OR “alpha galactosidase A deficiency” | 5784 |
| 10 | “Ceramide Trihexosidase Deficiency” OR “Glycolipid lipidosis” OR “Hereditary Dystopic Lipidosis” | 8 |
| 11 | #1 OR #2 OR #3 OR #4 OR #5 OR #6 OR #7 OR #8 OR #9 OR #10 | 13,910 |
| 12 | “Cost” AND (effective* OR efficien* OR utilit* OR minimi* OR consequen* OR benefit* OR unit* OR estimate* OR variable*) | 422,510 |
| 13 | “budget impact analysis” OR “budget impact model” OR (“budget impact” AND (method* OR analys* OR model* OR simulation* OR assessment*)) OR “BIA”[Title/Abstract] | 5656 |
| 14 | “economic evaluation” OR “economic model” OR “pharmacoeconomics” | 21,765 |
| 15 | “markov” AND (method* OR analys* OR model* OR simulation* OR assessment* OR chain*) OR “hidden markov model” OR “Markov chain” | 30,544 |
| 16 | “monte carlo method” OR (“monte carlo” AND (method* OR analys* OR model* OR simulation* OR assessment* OR chain*)) OR “Monte Carlo simulation” OR “Monte Carlo technique” | 61,663 |
| 17 | “cost effectiveness analysis” OR ((“cost effectiveness” OR “cost effective”) AND (method* OR analys* OR model* OR simulation* OR assessment*)) OR “cost effectiveness ratio” OR “cost effectiveness” OR “cost-effectiveness” OR “CEA”[Title/Abstract] OR “CER”[Title/Abstract] | 164,700 |
| 18 | “cost efficiency analysis” OR (“cost efficiency” AND (method* OR analys* OR model* OR simulation* OR assessment*)) OR “cost efficiency” OR “cost-efficiency” | 2537 |
| 19 | “cost benefit analysis” OR (“cost benefit” AND (method* OR analys* OR model* OR simulation* OR assessment*)) OR “cost benefit” OR “cost-benefit” OR “cost benefit ratio” OR “cost-benefit ratio” OR “CBA” OR “CBR” | 126,024 |
| 20 | “cost utility analysis” OR ((“cost utility” OR “cost utilities”) AND (method* OR analys* OR model* OR simulation* OR assessment*)) OR “cost utility” OR “cost-utility” OR “CUA” | 8168 |
| 21 | “cost minimization analysis” OR ((“cost minimization” OR “cost minimisation”) AND (method* OR analys* OR model* OR simulation* OR assessment*)) OR “cost minimisation” OR “cost minimization” OR “cost-minimisation” OR “cost-minimization” OR “CMA” | 7438 |
| 22 | “cost consequence analysis” OR (“cost consequence” AND (method* OR analys* OR model* OR simulation* OR assessment*)) OR “cost consequence” OR “cost- consequence” OR “CCA” | 11,069 |
| 23 | ((“cost” OR “economic”) AND (method* OR analys* OR model* OR simulation* OR assessment*)) OR “Health economic” AND stud* OR “decision analytic” OR “decision-analytic” | 379,777 |
| 24 | (“decision tree” OR “decision trees”)[Title/Abstract] | 19,783 |
| 25 | #12 OR #13 OR #14 OR #15 OR #16 OR #17 OR #18 OR #19 OR #20 OR #21 OR #22 OR #23 OR #24 | 789,553 |
| 26 | “economics” OR “economic aspect” OR “cost” OR “health care cost” OR “drug cost” OR “hospital cost” OR “socioeconomics” OR “health economics” OR “fee” OR “budget” OR “hospital finance” OR “financial management” OR “health care financing” OR “low cost” OR “high cost” OR health*care AND cost* OR “health care” AND cost* OR “fiscal” OR “funding” OR “financial” OR “finance” OR (“unit” AND cost*) OR price* OR “pricing” OR Expenditure* | 1,358,133 |
| 27 | Econ* AND (“Burden” OR “disease” OR “assessment”) OR Cost* AND (illness* OR health* OR “burden” OR “disease” OR “assessment”) | 642,154 |
| 28 | (“Out” AND “Pocket”) OR (Patient* AND Cost*) OR copay* OR (Privat* AND Expendit*) OR ((Carer* OR Caregiv*) AND (Cost* OR Expendit* OR Time)) | 372,097 |
| 29 | “Value” AND (“Money” OR “Monetary”) OR (cost* AND (treat* OR therap*)) | 361,420 |
| 30 | “Costly” OR “Costing” OR pharmacoeconomic* OR pharmaco-economic* OR “Finances” OR “Financed” OR “cost analysis” OR “cost assessment” OR “cost study” | 119,232 |
| 31 | “societal cost” OR “social cost” OR “social care cost” OR “out of pocket” OR “patient cost” OR “co-payment” OR “private expenditure” OR “patient time” OR “carer cost” OR “carer expenditure” OR “carer time” OR “caregiver cost” OR “caregiver expenditure” OR “caregiver time” OR “economic burden” OR “cost burden” OR “resource burden” OR “financial burden” OR “economic consequences” OR “cost of illness” OR “healthcare cost” OR “cost of disease” | 69,833 |
| 32 | “deductibles” AND “coinsurance” | 1894 |
| 33 | “value” AND “money” | 3686 |
| 34 | (health*care AND (utilisation OR utilization)) OR (“health care” AND (utilisation OR utilization)) OR (“resource” AND (utilisation OR utilization OR use)) | 438,460 |
| 35 | “resource use” OR “healthcare resources” OR “resource utilization” OR “resource” OR “health resource” OR “healthcare resource” | 248,683 |
| 36 | “Patient readmission” OR “Patient admission” OR “Length of stay” OR readmi* OR rehosp* OR “Hospital readmission” OR “Reoperation” OR “Emergency room” | 322,188 |
| 37 | (“Stay” AND (“Length” OR “Duration”)) | 168,059 |
| 38 | ((Outpatient* OR “clinic” OR physician* OR “office” OR specialist* OR “professional” OR “practitioner”) AND (visit* OR “care”)) | 703,778 |
| 39 | ((“resource” OR “staff”) AND (utilis* OR “allocation”)) | 28,431 |
| 40 | ((“high dependency” OR “intensive”) AND (“unit” OR “care”)) | 332,401 |
| 41 | “Resource allocation” OR “Healthcare utilization” | 24,269 |
| 42 | (“healthcare” OR “health care” OR service* OR resource* OR hospital* OR “clinic” OR “clinics”) AND (“visits” OR “utilisation” OR “utilization” OR “frequency” OR “number” OR “access”) AND (“patient” OR “patients” OR “parents” OR “subjects” OR “elderly” OR “adults”) | 827,188 |
| 43 | “health care rationing” OR “medical savings accounts” OR “resource allocation” | 28,159 |
| 44 | #26 OR #27 OR #28 OR #29 OR #30 OR #31 OR #32 OR #33 OR #34 OR #35 OR #36 OR #37 OR #38 OR #39 OR #40 OR #41 OR #42 OR #43 | 3,537,940 |
| 45 | #25 OR #44 | 3,742,815 |
| 46 | #11 AND #45 | 1064 |
| 47 | #46 AND (in process[sb] OR pubstatusaheadofprint)) | 16 |
| 48 | #46 AND (in process[sb] OR pubstatusaheadofprint)) Filters: from 2019/12/20 - 2022/1/18 | 16 |

| **EconLit** | | **Search hits** |
| --- | --- | --- |
| **Disease facet (Fabry disease)** | | |
| 1 | ‘Fabry Disease’ OR ‘Fabry’ OR ‘angiokeratoma corporis diffusum’ OR ‘anderson-fabry disease’ OR ‘anderson fabry disease’ OR ‘Galactosidase deficiency’ OR ‘Ceramide Trihexosidase Deficiency’ OR ‘Glycolipid lipidosis’ OR ‘Hereditary Dystopic Lipidosis’ | 0 |

| **NHS EED database produced by the CRD** | | **Search hits** |
| --- | --- | --- |
| 1 | (‘Fabry Disease’ OR ‘Fabry’ OR ‘angiokeratoma corporis diffusum’ OR ‘anderson-fabry disease’ OR ‘anderson fabry disease’ OR ‘Galactosidase deficiency’ OR ‘Ceramide Trihexosidase Deficiency’ OR ‘Glycolipid lipidosis’ OR ‘Hereditary Dystopic Lipidosis’) IN DARE, NHSEED, HTA | 0 |

**Supplementary Table 2.** Eligibility criteria for both reviews

|  | Eligibility criteria |
| --- | --- |
| Patient age | Adults and children (adult patients and children with a confirmed diagnosis of Fabry disease are of interest) |
| Sex | Both male and female patients/controls |
| Race | Any |
| Ethnicity | Any |
| Intervention/ Comparator | No restriction on intervention or comparator |
| Language | English language only |
| Publication timeframe | No restriction on the publication timeframe. Database inception to January 19, 2022 |

**Supplementary Table 3.** Description of the instrument/tools utilized to assess QoL across the studies included in the humanistic search

| **Instrument/tool** | **Description** | **Interpretation of scores** | **Number of studies** | **Study reference** |
| --- | --- | --- | --- | --- |
| ***Health-related QoL*** | | | | |
| SF-36 | A generic instrument most frequently used to measure overall HRQoL. Thirty-six items cover eight domains of physical, psychological, and social functioning. “Physical Functioning” (PF) scores patients’ performance related to daily activities; “Role- Physical” (RP) regards the impact of physical health on life; “Bodily Pain” (BP) evaluates the pain level and its impact on normal daily activities; “General Health” (GH) evaluates subjective perception about the present and future health status and resistance to illness; “Vitality” (VT) scores patients’ feelings about their energy, vitality and moments of fatigue; “Social Functioning” (SF) scores the impact of health on routine social activities; “Role Emotional” (RE) measures the influences of emotional status on daily activities; and “Mental Health” (MH) scores mood and well-being, including depression and anxiety | Each domain is scored from 0 (worst) to 100 (best) | 16 | (19, 26, 30-41, 43, 44) |
| SF-12 | A 12-item version of the Short Form survey, designed to reduce the burden of patient respondents whilst maintaining minimum precision standards for the purposes of comparisons between groups across multiple health dimensions. The survey includes Physical and Mental Component Summary scores | Each domain has a mean of 50 and SD of 10 | 1 | (42) |
| EQ-5D descriptive system | The tool comprises the following five dimensions: Mobility, Self-Care, Usual Activities, Pain/Discomfort, and Anxiety/Depression | Each dimension has three levels: either no problems, some/moderate problems, extreme problems or any problem, moderate problems, extreme problems | 5 | (35, 41, 45-47) |
| EQ-5D VAS | The tool comprises the following five dimensions: Mobility, Self-Care, Usual Activities, Pain/Discomfort, and Anxiety/Depression | The QoL rated on a scale of 0 (worst imaginable health status) to 100 (best imaginable health status) | 7 | (33-35, 41, 46, 48, 49) |
| PedsQL | The 23-items PedsQL 4.0 instrument comprises comprise four generic core scales i.e., physical functioning (eight items), emotional functioning (five items), social functioning (five items), and school functioning (five items). Further, two summary scores (psychosocial health summary score [mean of 15 items – five items each of emotional, social, and school functioning], and the physical health summary score [the same as eight items of the physical functioning]) and the total score (23 items) were also computed. A five-point Likert response scale is utilized to rate each item (0 = never a problem; 1 = almost never a problem; 2 = sometimes a problem; 3 = often a problem; 4 = almost always a problem) | The scale ranged from 0 to 100, where higher scores indicate better HRQoL | 2 | (20, 40) |
| Author’s questionnaire | An author’s disease-specific questionnaire was developed based on the literature, personal experiences, patient-related observations, and patient-collected information. The questionnaire consists of 25 questions and four parts: Part 1: The first five questions concern personal details Part 2: It includes six closed questions with conjunctive choices, aimed at identifying the health problems that generate a significant impact on the HRQoL. The respondents were also asked to evaluate their health status in both the physical and mental sense Part 3: The subsequent nine questions were directed at patients undergoing enzymatic replacement therapy (ERT). The respondents were asked to state their opinion whether the ERT produced any effects on their current health condition, and if so, in relation to which symptoms. The questionnaire contained eight closed questions and one open question where the respondents were asked to indicate in relation to which symptoms they expected more significant health improvement Part 4: This part determines whether patients receive support from their families and community and to investigate the importance of the patients’ organizations, in this case the “Association of Fabry Families”. The respondents were asked five questions, four closed and one question with half open choices, including a set of responses with an additional item “others” in case that the response that is incompatible with any of the provided options | The health status was either rated as excellent, very good, good, quite good, and bad or Big, substantial, slight, very slight, and none | 1 | (41) |
| Patient health and lifestyle questionnaire | It comprises four general categories: general symptoms, organ involvement, quality of life, and treatment with ERT | Not reported | 1 | (51) |
| WHO QoL-100 | A generic, patient-completed measure of HRQoL comprises six domains, namely Physical, Psychological, Independence, Social relationships, Environment, and Spirituality. These domains further consist of a total of 24 facets; each facet includes four items which pertain to the general quality of life value. Each of the facets is summed and each item contributes equally to the facet score and the domain score | All the answers of the patients are punctuated by a five-point Likert scale (higher scores indicate better QoL values) | 1 | (93) |
| BASC-2 | The BASC-2 included 16 items which measure adaptive and maladaptive functioning in emotional and behavioral domains. The parents rank each item from “never occurs” to “almost always occurs” describing child behavior. The Externalizing Problems Composite scale measures hyperactivity, aggression, and conduct problems. The subscales measuring internalized problems are anxiety, depression, and somatization | The parents rank each item from “never occurs” to “almost always occurs”, describing child behavior | 1 | (20) |
| Health States and QoL | Association of health states and quality of life | Not reported | 2 | (45, 46) |
| RAND 36-Item Health Survey questionnaire | A validated tool to assess the physical, mental, and social aspects of HRQoL. It comprises eight HRQoL domains: Physical functioning, Role limitations due to physical health, Role limitations due to emotional problems, Energy/fatigue, Emotional well-being, Social functioning, Pain, and General health. Each domain represents a different aspect of health status and a separate question also measures perceived health changes | Items in each domain ranged from 0 to 100, with 0 being the worst and 100 being the best | 1 | (95) |
| ***Pain*** | | | | |
| BPI | The tool has been designed to assess the severity of pain and the impact of pain on daily functions. The latter is reflected by the BPI interference score, which is the average of the following interference subscales: General activity, Mood, Walking ability, Normal work, Relations with other people, Sleep, and Enjoyment of life | The subscales are scored from 0 to 10, with an estimated minimally important difference of 1 or 0.5 SD | 8 | (32, 33, 37, 39, 45, 46, 49, 52) |
| VAS for Pain | Not reported | No pain (score 0) to maximal pain (score 10) | 2 | (38, 53) |
| NRS for Pain | A brief questionnaire was developed by the authors to measure FD symptomatology (including pain). The questionnaire consisted of five-point Likert rating scales and yes/no questions focused on patient perceptions of Fabry-related pain, including quantitative descriptors of the neuropathic pain (e.g., intensity, frequency), and quality of life | Five-point numeric rating scales were used to assess FD pain intensity and pain unpleasantness | 1 | (54) |
| Joint Pain Questionnaire | The “Joint Pain Questionnaire” included ten general questions on the presence of musculoskeletal problems, as well as parts of established and validated questionnaires, e.g., EQ-5D, BPI, Patient’s assessment of physical function (HAQ-DI), SF-36 Health Survey, and McGregor’s questionnaire | Not reported | 1 | (55) |
| ***Mental Health*** | | | | |
| CES-D | CES-D-4 items scale comprises four-items. Each item assesses emotional symptoms of depression; no somatic symptoms are included in the scale. The response to each item was rated on a scale of 0 to 3  CES-D-20 items is a self-reporting scale and was utilized to assess the frequency of symptoms during the previous week | The total scores range from 0 to 12, with higher scores denoting more depressive symptoms. Further, a score of 4 or higher suggests a clinically significant level of psychological distress  A total score ranged from 0 to 60. Scores between 15 to 21 indicate “mild to moderate” depressive symptoms, scores above 21 indicate “severe” depressive symptoms | 2 | (42, 56) |
| BDI-II | BDI-II questionnaire is utilized to determine depression symptoms and severity, and contains 21 questions. Each question is answered on a four‐point scale with the value from 0 (not at all) to 3 (an extreme form of each symptom) | The total score ranged from 0 to 63 with higher scores indicate more severe depressive symptom | 1 | (33) |
| CDI | CDI-2 is a 28-item self-reported assessment tool that assesses cognitive, affective, and behavioral signs of depression in children and adolescents 7 to 17 years old. The CDI-2 measures symptoms of depression based on a total score, two scale scores, and four subscales | Each item is rated on a scale of 0–2 that correspond to three levels of symptomatology: 0 (absence of symptoms), 1 (mild or probable symptom), or 2 (definite symptom) | 1 | (20) |
| HAM-D | HAM-D 17 is a 17-item scale to measure the severity of depressive symptoms. Each item is scored on a four-point-scale from 0 (not present) to 4 (severe).  An expanded version of the scale, HAM-D 21 includes an additional four items that may be related to depression, but not severity, such as paranoia and obsessive or compulsive symptoms. | The total score for HAM-D 17 ranges from 0 to 52, with higher scores denoting more severe depressive symptoms. Scores between 0 and 7 are considered normal, scores between 8 and 16 suggest mild depression, scores between 17 and 23 suggest moderate depression, and scores above 24 are indicative of severe depression. | 1 | (57) |
| HADS | Not reported | Not reported | 1 | (49) |
| PSS-4 | PSS-4 items questionnaire elicits perceptions of stress during the past month | The response to each item is assigned a value of 0 to 4; a total score ranged from 0 (no stress) to 16 (high stress) | 1 | (42) |
| **Sleep and fatigue** | | | | |
| ESS | The ESS is a self-administered questionnaire with eight questions. Each question is to be rated on a four-point scale (0–3) to judge the patient’s usual chances of dozing off or falling asleep while engaged in different activities. The eight activities are 1) Sitting and reading; 2) Watching TV; 3) Sitting inactively in a public place; 4) As a passenger in a car for an hour without a break; 5) Lying down to rest in the afternoon when circumstances permit; 6) Sitting and talking to someone; 7) Sitting quietly after lunch without alcohol; and 8) In a car while stopped for a few minutes in traffic. | The ESS score (the sum of 8 item scores) can range from 0 to 24. The higher the ESS score, the higher that person’s average sleep propensity in daily life (ASP), or their ‘daytime sleepiness’ | 2 | (19, 33) |
| RBDSQ | This is a patient self-rating instrument with 10-items, to be answered either yes or no, which assesses various aspects of sleep behavior. Items 1 to 4 assess the content and frequency of dreams and their relationship to nocturnal movements and behavior; item 5 asks about potential nocturnal injuries sustained by the patient or their bed partner; item 6 is divided into four subsections and is designed to assess nocturnal motor behavior (for example, vocalization, sudden limb movements, complex movements, or bedding items that fall from the bed); items 7 and 8 deal with nocturnal awakenings; item 9, with disturbed sleep in general; and item 10, with the presence of any neurological disorder | The maximum total score obtained is 13, with a higher score suggesting more features associated with RBD | 1 | (33) |
| FSS | Not reported | Not reported | 1 | (58) |
| CoL questionnaire | This is used to assess psychosocial development. It comprises five scales, namely ‘Autonomy development’ (six items, autonomy at home and outside the home; score 6–12), ‘Psychosexual development’ (four items, love and sexual relations; score 4–8), ‘Social development’ (12 items, contacts with peers; score 12–24), ‘Antisocial behavior’ (four items, misbehavior at school and outside school; score 4–8) and ‘Substance use and gambling’ (12 items; score 12–24) | A higher scale score on the ‘Autonomy development’, ‘Psychosexual development’, and ‘Social development’ indicates the accomplishment of more psychosocial developmental milestones | 1 | (30) |
| ASEBA | The ASEBA questionnaire for adults was designed to assess social-adaptive and maladaptive functioning using Diagnostic and statistical manual of mental disorders IV (DSM IV)-oriented scales for subjects aged between 18 years to 59 years. ASEBA forms comprise the raw data from the adult self-report questionnaire (ASR) (208 self-assessed questions which are completed by the primary subject) and adult behavior checklist (ABCL) (152 questions completed by a close family member or friend, about the patient’s functioning). The report from ASEBA included scores from the DSM-oriented scales for depressive problems and anxiety problems, as well as from an adaptive functioning scale, which assesses social functioning (ASR, ABCL), and occupational functioning (ASR only). Scale scores were normalized by gender and age group. The mean scores for adaptive functioning were calculated for all patients who had completed an ASR and then classified as normal (below the 93rd percentile), borderline clinical (93rd to 97th percentiles), or clinical (above the 97th percentile) for the DSM IV diagnostic categories. The patients with borderline clinical or clinical adaptive functioning deficit were considered to have a social-adaptive functioning deficiency (SAFD) | Not reported | 1 | (94) |

ASEBA, Achenbach System of Empirically Based Assessment; BASC-2, Behavior Assessment System for Children 2nd Edition; BDI-II, Beck Depression Inventory-II; BPI, Brief Pain Inventory; CDI, Children’s Depression Inventory; CES-D, Center for Epidemiologic Studies Depression Scale; CoL, course of life; ESS, Epworth Sleepiness Scale; ERT, enzyme replacement therapy; FD, Fabry disease; FSS, Fatigue Severity Scale; HADS, Hospital Anxiety and Depression Scale; HAM-D, Hamilton Rating Scale for Depression; HRQoL, health-related quality of life; NRS, Numerical Rating Scale; PedsQL, Pediatric Quality of Life Inventory; PSS-4, Perceived Stress Scale – 4 items; QoL, quality of life; RBD, rapid eye movement sleep behavior disorder; RBDSQ, Rapid Eye Movement Sleep Behavior Disorder Screening Questionnaire; SF-12, 12-item Short-Form Health Survey; SF-36, 36-item Short-Form Health Survey; VAS, visual analog scale; WHO, world health organization.

**Supplementary Table 4.** Summary of findings from additional general HRQoL tools used in only one study

| **Study** | **Instrument/Tool** | **Key findings** |
| --- | --- | --- |
| Street *et al.* (95) | RAND-36 | Significantly lower scores (*p* < 0.0001) across all subscales (covering physical, mental and social aspects of HRQoL) in women with FD when compared with the control group |
| Cazzorla *et al*. (93) | WHO QoL-100 | Mean (SD) general QoL score of 12 (3.7) reported in patients with FD; lowest score observed in the environmental domain (mean [SD]: 7.9 [1.7]) and highest score in the physical domain (mean [SD]: 13.2 [2.0]) |
| Bouwman *et al*. (30) | CoL Questionnaire | Young adult male patients with FD achieved significantly fewer milestones in social development than male controls (*p*= 0.02); there was no significant difference in achievement of social milestones between young adult female patients with FD and controls |
| Zuraw *et al*. (41) | Author’s Questionnaire | Patients with FD perceived that burning extremity pain, gastrointestinal disorders, and abdominal renal function had the biggest impact on their quality of life. An improvement in symptoms was perceived following ERT for at least 50% of the patients in each symptom category |
| Morier *et al*. (51) | Patient Health and Lifestyle Questionnaire | The majority of patients reported some degree of impact of FD on their quality of life, with a higher proportion of men reporting an impact than women (87.5% vs 60%, respectively); the proportion of patients reporting that their life is greatly impacted was similar between men and women (12.5% vs 13.3%, respectively) |
| Bugescu *et al*. (20) | BASC-2 | Parents of children aged 12–18 years reported significantly higher scores for their children in internalizing problems (including anxiety, depression and somatization), attention problems, and adaptability compared with a normative population; no significant differences to the normative population were reported for children aged 6–11 years |
| Laney *et al. (94)* | ASEBA | A mean adaptive score within the clinical range of SAFD was reported for 26.7% of patients with FD; statistically significant relationships were identified between SAFD and several concomitant conditions including depression (*p* < 0.01), antisocial personality (*p* < 0.001), attention-deficient/hyperactivity disorder (*p* < 0.01), and hyperactivity-impulsivity ( *p* <0.01) |

ASEBA, Achenbach System of Empirically Based Assessment; BASC-2, Behavior Assessment System for Children 2nd Edition; CoL, course of life; ERT, enzyme replacement therapy; FD, Fabry disease; HRQoL, health-related quality of life; QoL, quality of life; SAFD, social-adaptive functioning deficiency; SD, standard deviation
